# Untangling the Interplay between Epidemic Spread and Transmission Network Dynamics

**DOI:** 10.1371/journal.pcbi.1000984

**Published:** 2010-11-18

**Authors:** Christel Kamp

**Affiliations:** Biostatistics, Paul-Ehrlich-Institut, Federal Institute for Vaccines and Biomedicines, Langen, Germany; Imperial College London, United Kingdom

## Abstract

The epidemic spread of infectious diseases is ubiquitous and often has a considerable impact on public health and economic wealth. The large variability in the spatio-temporal patterns of epidemics prohibits simple interventions and requires a detailed analysis of each epidemic with respect to its infectious agent and the corresponding routes of transmission. To facilitate this analysis, we introduce a mathematical framework which links epidemic patterns to the topology and dynamics of the underlying transmission network. The evolution, both in disease prevalence and transmission network topology, is derived from a closed set of partial differential equations for infections without allowing for recovery. The predictions are in excellent agreement with complementarily conducted agent-based simulations. The capacity of this new method is demonstrated in several case studies on HIV epidemics in synthetic populations: it allows us to monitor the evolution of contact behavior among healthy and infected individuals and the contributions of different disease stages to the spreading of the epidemic. This gives both direction to and a test bed for targeted intervention strategies for epidemic control. In conclusion, this mathematical framework provides a capable toolbox for the analysis of epidemics from first principles. This allows for fast, *in silico* modeling - and manipulation - of epidemics and is especially powerful if complemented with adequate empirical data for parameterization.

## Author Summary

The way potentially infectious contacts are made strongly influences how fast and how widely epidemics spread in their host population. Therefore, it is important to assess changes in contact behavior throughout an epidemic; these may occur due to external factors, such as demographic change, or as a side effect of the epidemic itself, leading to an accumulation of individuals with risky behavior in the infected population. We have developed a mathematical framework that allows for the study of the mutual interdependencies between epidemic spread and changes in contact behavior. The method is used to study HIV epidemics in model populations. We address the question of whether HIV is primarily spread by highly contagious initially infected hosts or by less contagious latently infected hosts who will, however, encounter more situations for potential transmission. The answer to this question strongly depends on the concurrency of contacts and the maturation stage of the epidemic. Initially infected hosts are major epidemic spreaders in populations with strongly concurrent contacts and during epidemic expansion whereas otherwise latently infected hosts play a more important role. The availability of better data for parameterization will make this approach relevant for public health considerations.

## Introduction

Despite huge efforts to improve public health, the spread of infectious diseases is still ubiquitous at the beginning of the 21st century, and there is considerable variability in epidemic patterns between locations. Although the recent influenza pandemic has been a global challenge, there have nonetheless been differences in its timing in the northern and southern hemisphere due to seasonal effects [1], [2]. Another prominent example for epidemic variability is the prevalence of sexually transmitted diseases (STDs), specifically HIV infections. Although HIV is endemic in many populations at low levels or restricted to high-risk groups, it has become highly endemic in other parts of the world [3], [4]. As a consequence, the spread of infectious diseases cannot be understood globally but understood only as the result of several local factors, such as climate and hygiene conditions, population density and structure, and cultural habits and mobility. Epidemic models aim to capture the mechanisms that link these factors to the emergent epidemics and to promote an understanding of the underlying dynamic processes as a prerequisite for intervention strategies [5], [6]. A useful abstraction in this context is to regard individuals that may be infected as nodes of a network in which the links are the potentially infectious contacts among individuals (or nodes in network notation). A major remaining challenge in modern epidemiology is to link the variability of transmission networks to the corresponding emergent epidemics.

Models that are flexible and can be adapted to specific epidemic situations best meet these challenges. Because we focus on the interplay between transmission network topology and epidemics, we will restrict ourselves to diseases caused by agents that lead to either immunity or death in their host, i.e., in which infection can occur only once. These epidemics can be described by Susceptible-Infected-Recovered or SIR models [5], [6]. We refer to the mathematically closely related case, where infection eventually leads to the death of the host, as a SID model (Susceptible-Infected-Death). The original, or classical, SIR model [7] assumes a mass-action type dynamic and as a consequence describes epidemics in homogeneous, well-mixed populations. Because this is generally not a good approximation of real world situations, current epidemic models strive for an integrated approach that considers both information about the course of disease (i.e., susceptible, infected and recovered stages) and the relevant transmission network [8], [9]. The models vary in their assumptions, attention to detail, computational costs, and as a consequence, their fields of application. Compartmental SIR models consider different contact patterns in sub-populations and link them via a contact matrix [10], providing a coarse-grained, but often adequate, representation. Network-based SIR models consider the distribution in each individual's number of infectious contacts 
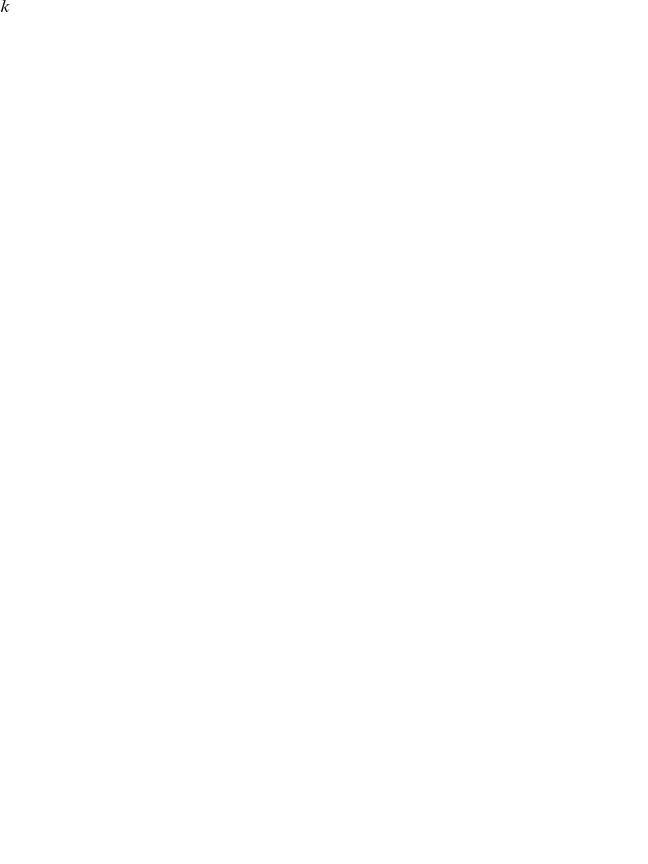
 in the transmission network (i.e. each node's degree 
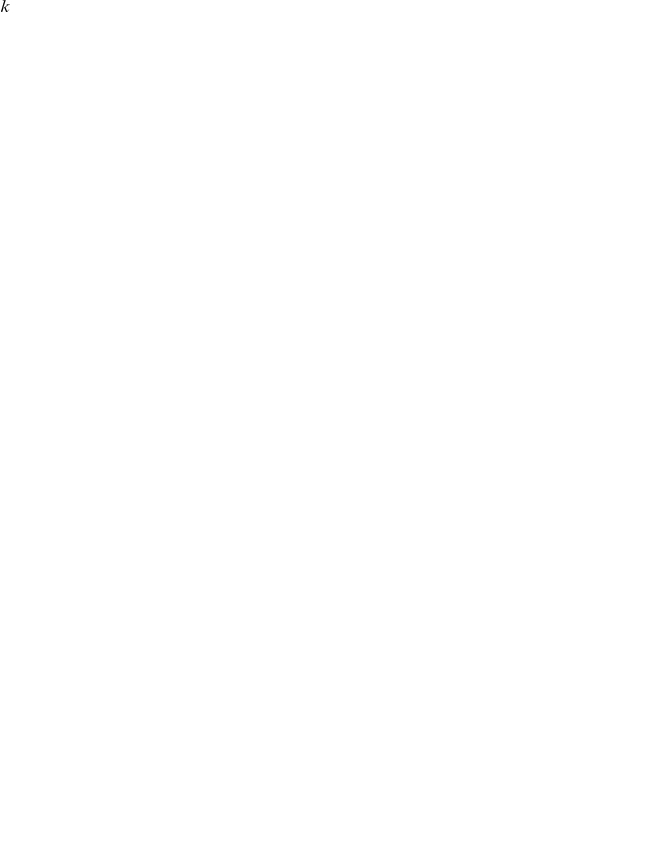
 in network notation) [11]–[13]. These models allow for the study of transmission networks with strong heterogeneity in the number of contacts among individuals, which in some cases also means that they consider correlations in the way contacts are made [14], [15], or clustering [16]–[18]. Although these approaches focus on static networks, a recent approach considers networks with arbitrary degree distributions and transient contacts and allows for the derivation of the temporal evolution in the number of susceptible and infected nodes from a closed set of equations [19]–[21]. Finally, pair models are a very general approach [22] for studying SIR epidemics on heterogeneous networks. They provide a large amount of flexibility in considering the way contacts are made (correlations or clustering) and maintained [23]–[25], but, as a trade-off, they quickly become very computationally demanding [26].

An assumption often implicitly made in epidemic models is that the epidemic sweeps through the population at much shorter time scales than the time scale of background demographic processes, i.e., natural birth and death processes are neglected. This is a good approximation in cases such as the yearly influenza epidemics, but it is hardly adequate for HIV epidemics, which span decades. To compensate for this limitation, we integrated demographic background processes into recent network epidemic models [19], [20]. With HIV in mind as a case study, we focus on disease epidemics that lead to death after infection of susceptible individuals, possibly after undergoing several stages of the disease. In addition to earlier work, our approach also allows for an in depth study of the interplay between epidemic spreading and the structure and dynamics of the underlying transmission network. Strictly speaking, all approaches discussed only predict the mean behavior of epidemics within the limit of an infinite host population. However, our comparisons with finite size, agent-based simulations show that this is a good and computationally efficient approximation already for moderate population sizes.

## Methods

To predict epidemic outcomes on the basis of the relevant transmission network, we study its components in more detail. The network's nodes represent a pathogen's hosts and the network's links are potentially infectious contacts among hosts. The mathematical framework is not restricted to any specific pathogen (and therefore route of transmission), so links or infectious contacts may represent quite different settings, for example sexual contacts in case of STDs or close spatial proximity in case of airborne infections. We will first focus on a simple SID model with one infectious stage, I, before death, D, and study its basic properties. This can naturally be extended to models with several stages of disease before death, for which we will show an example of the simplest case, a SI_1_I_2_D model with two stages of disease, I_1_ and I_2_, before death (See Text S1).

### The SID model

Assuming knowledge about infectious contacts, hosts (or nodes) can be assembled into subgroups according to their number of infectious contacts (or degree 
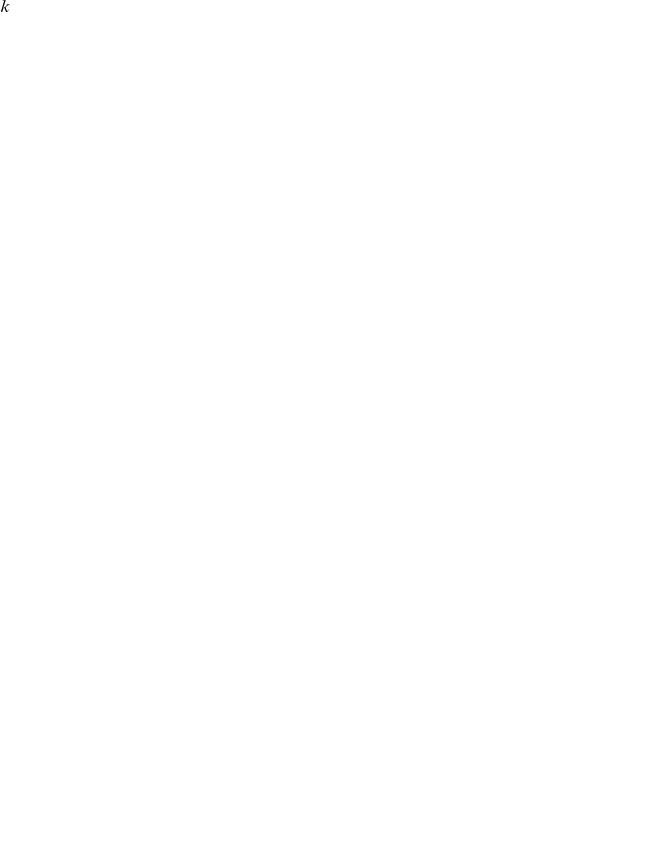
 in network notation). Following the ansatz of [19], [20], evolution equations for each of these subgroups are then derived which allows us to model arbitrary heterogeneity in the number of infectious contacts between hosts. Susceptible hosts get infected at a rate that is proportional to their number of contacts, 
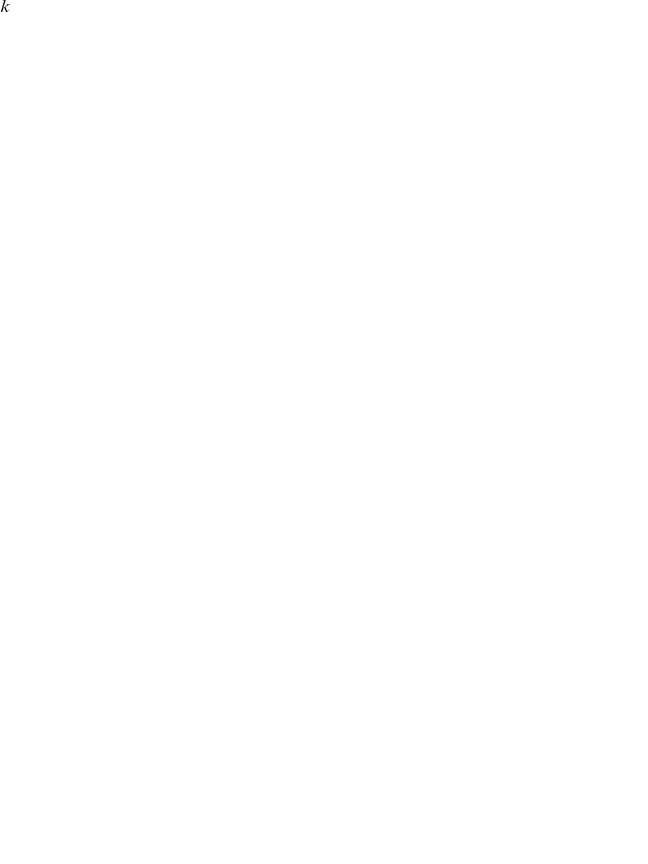
, the transmission rate per contact, 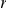, as well as the probability that a contact made by a susceptible individual links to an infected individual, 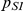. The number of hosts with a given number of contacts changes either by birth and death or by birth and death of the hosts' contacts, where death may occur both due to disease (at a rate 
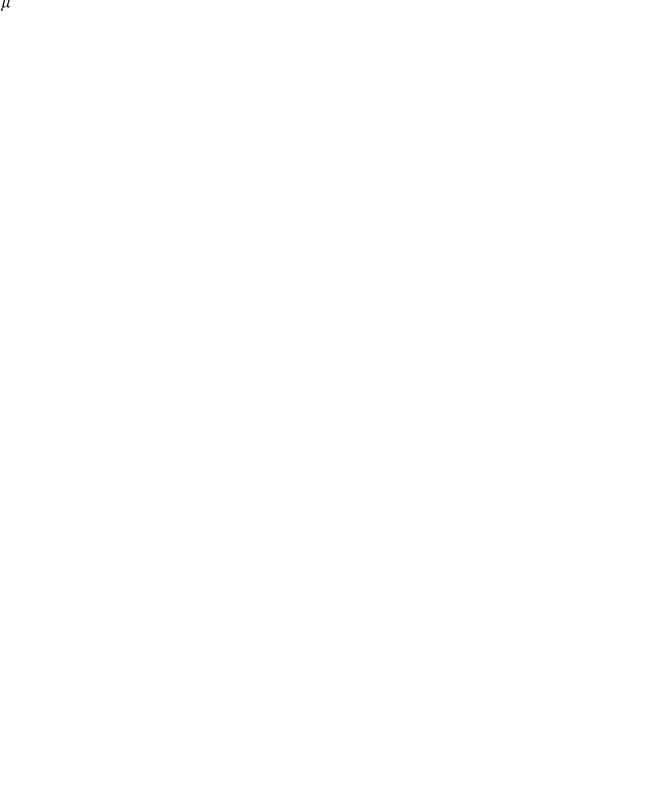
) or due to other causes (at a rate 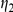). Individuals entering the population at a rate 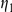 establish 
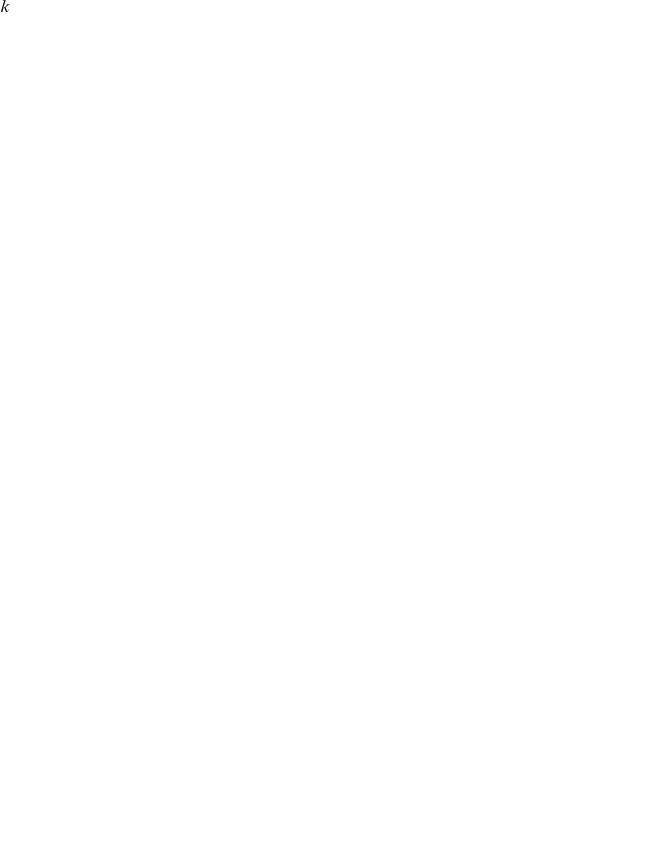
 contacts with probability 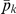 (corresponding to the probability generating function 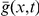).

Table 1 summarizes the parameters and notation of the model which allows us to compile the equations for the numbers of susceptible and infected individuals with 
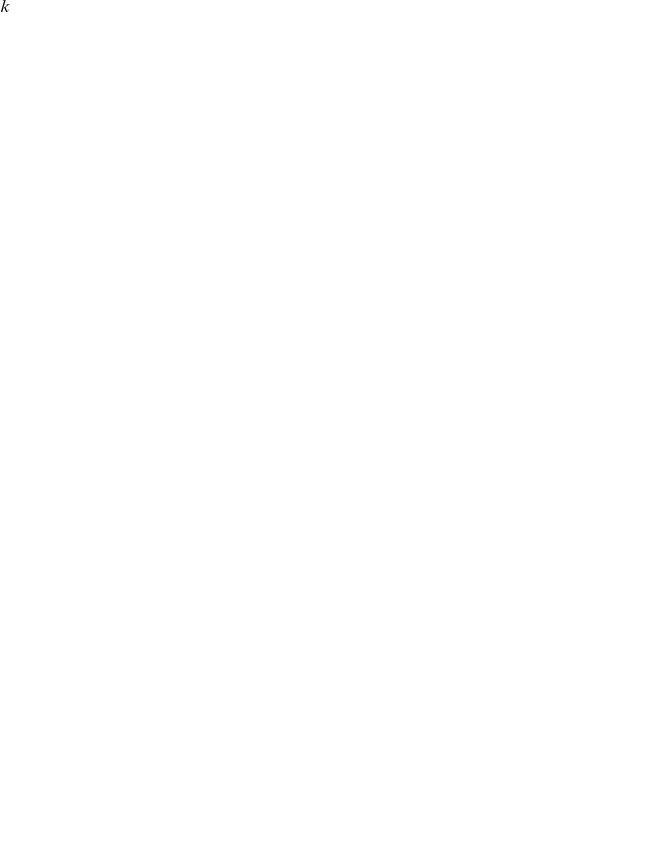
 contacts 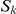 and 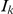 as:
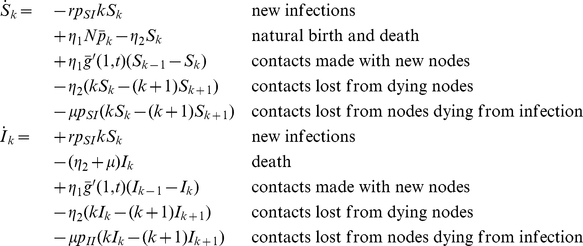



**Table 1:** Notation and parameters of the model.

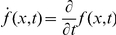	partial derivative of function 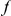 with respect to 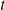
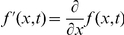	partial derivative of function 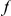 with respect to 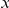
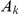	number of individuals in group 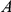 with 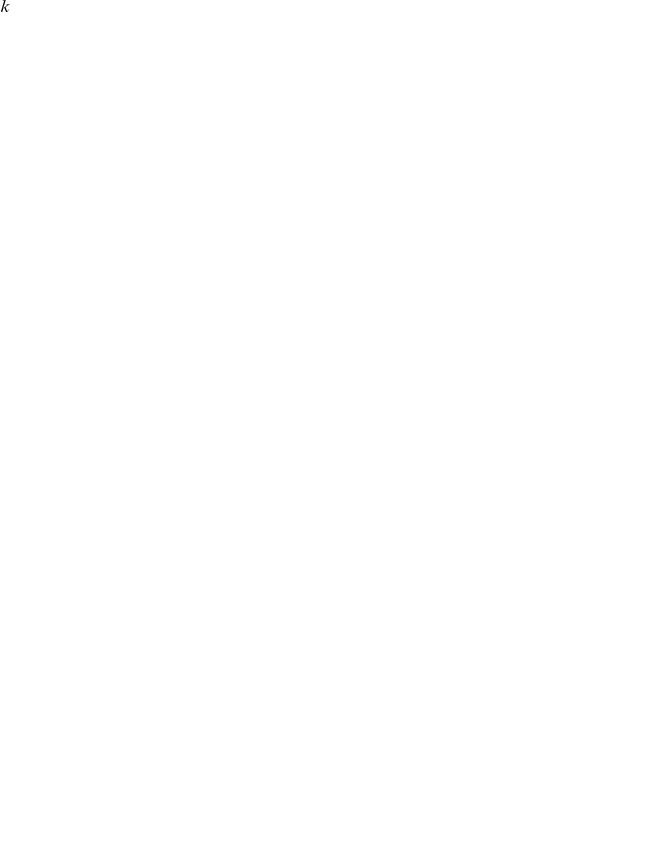 contacts
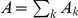	total number of individuals in 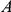
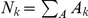	number of individuals with 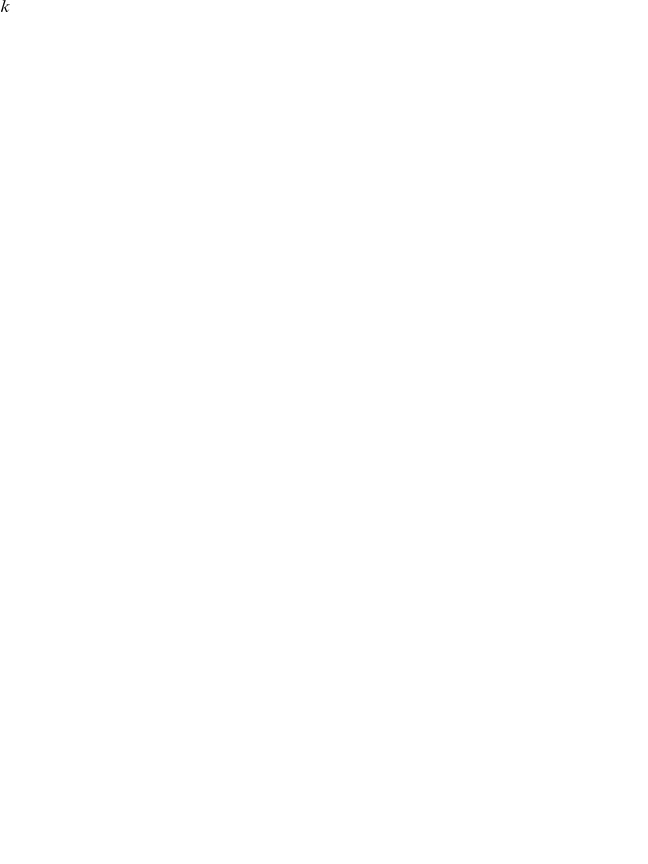 contacts
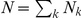	total number of individuals
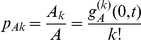	probability for an individual in group 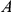 to have 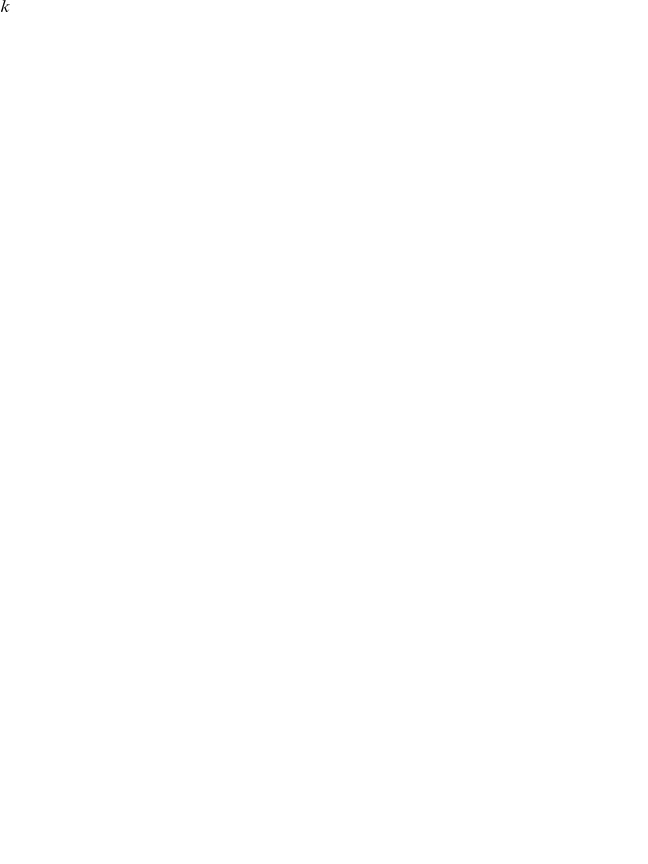 contacts
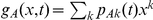	probability generating function (PGF) of 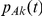
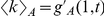	average number of contacts of 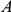 individuals
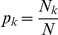	probability for an individual to have 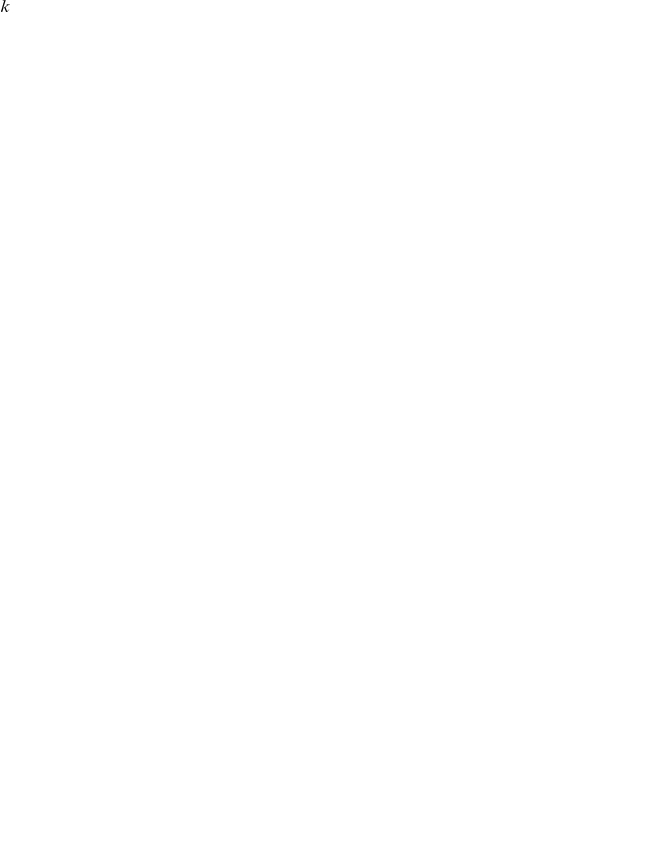 contacts
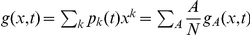	probability generating function (PGF) of 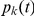
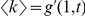	average number of contacts
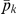	probability of a person entering the population to have 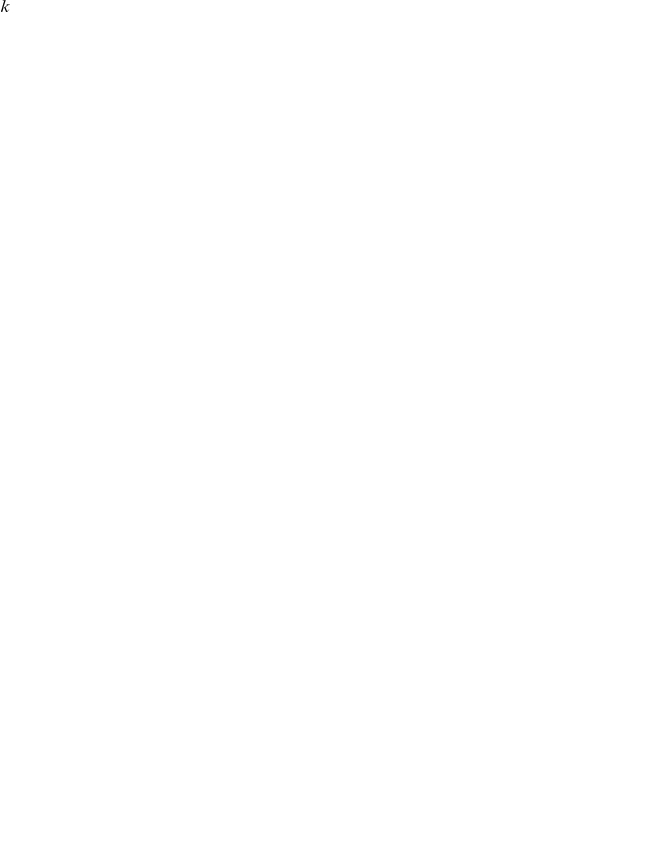 contacts
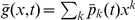	probability generating function (PGF) of 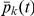
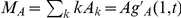	number of links emanating from 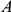 individuals
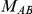	number of links emanating from 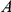 individuals and pointing to 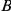 individuals
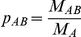	probability for a link starting from an 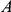 individual to point to a 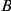 individual
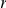 ( 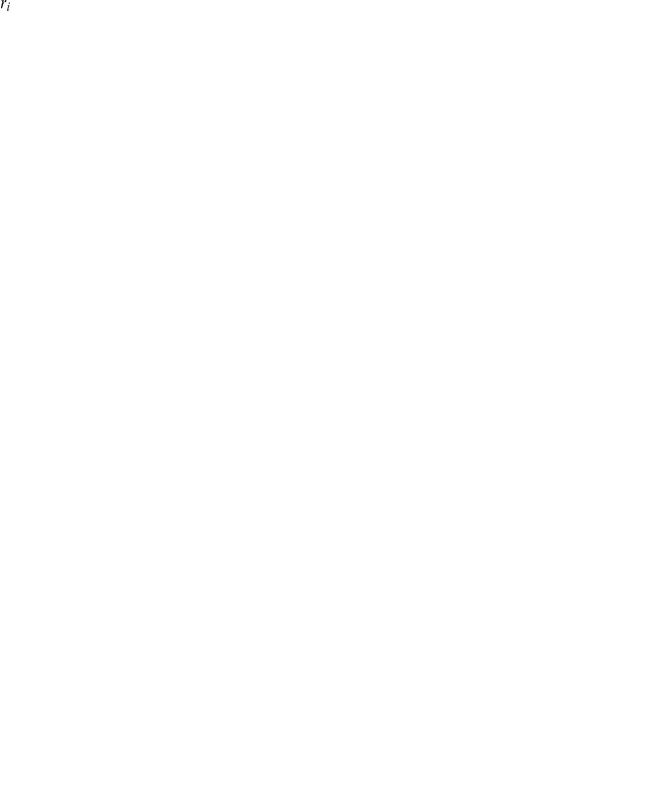 )	transmission rate per contact (at stage 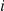 of infection)
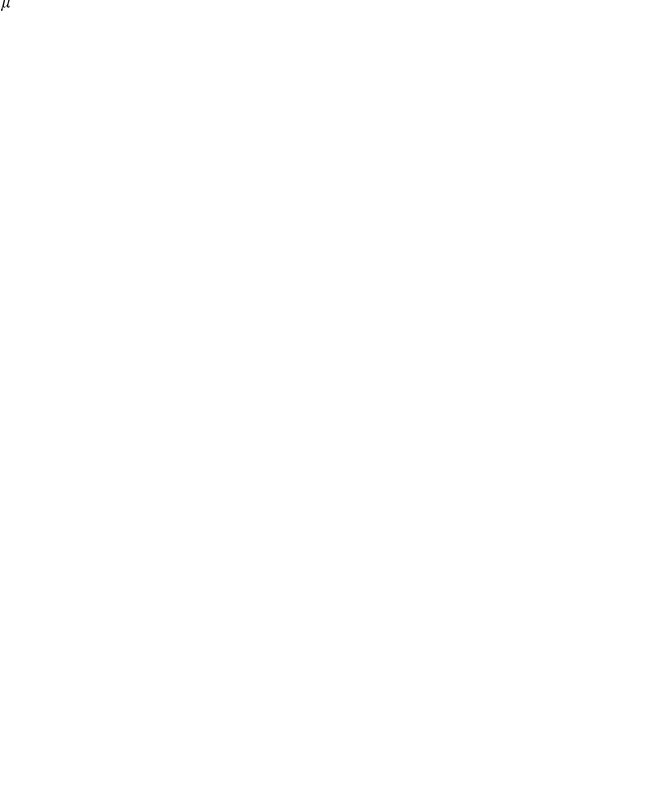 ( 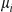 )	progression/death rate (at stage 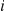 of infection)
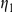 , 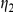	birth and death rate
Parameters for the HIV epidemic models [33]	
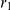 = 2.76 p.a.	transmission rate during primary infection (per year)
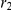 = 0.1 p.a.	transmission rate during latent infection (per year)
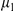 = 4.1 p.a.	progression rate of primary infection (per year)
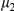 = 0.12 p.a.	progression rate of latent infection (per year)

Note, that 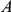, 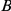 correspond to the stages that are passed during an infection, e.g. 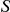 for susceptible, 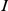 for infected etc. Throughout the manuscript derivatives with respect to time/spatial variables are denoted by a dot/prime.

Note that it is implicitly assumed that new individuals enter the population at a rate 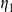 have 
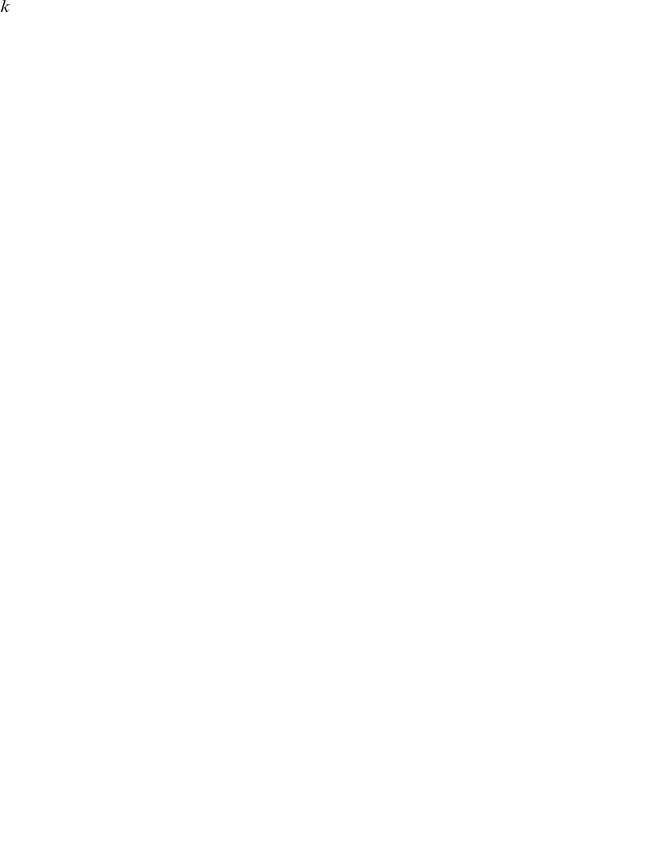
 contacts with probability 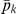 by which they link randomly to those individuals already present. All presented data are based on this dynamic for the establishment of new nodes' contacts, as this can be considered as the most basic dynamic model. However, the approach is not restricted to this case and can naturally be extended to other dynamics, for example preferential attachment of links with respect to the target nodes' degree (See Text S1). It is further assumed that each individual in the population of size 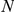 can give birth to a susceptible individual at the same rate 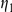 which may be conditioned on the individuals' health status in future models. Individuals dying from natural causes at a rate 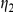 are assumed to have the same average number of contacts as found in the whole population without preferences for susceptible or infected individuals. In terms of the total number 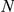 of susceptible 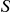 and infected individuals 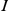 the equations read
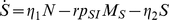
(1)

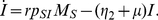
(2)To close this set of equations and to take into account local clustering of infectious cases, additional equations have to be derived for 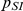 and 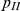, the probabilities that a contact made by a susceptible or infected individual points to an infected individual. We apply the techniques developed in [19] (See Text S1 for detailed calculations and discussion of the underlying approximations) and conclude
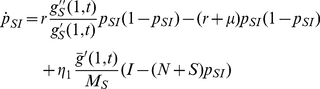
(3)

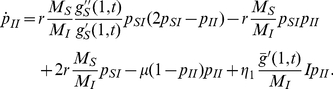
(4)


The occurrence of the probability generating functions (PGFs) in equations (3) and (4) which represent the degree distributions of susceptible and infected individuals makes it clear that the way links are maintained between susceptible and infected nodes depends on the contact behavior within these subgroups, as well as the changes therein in response to the epidemic. In other words, the set of equations can only be closed if the time evolution of the PGFs 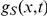 and 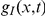 is considered. With 
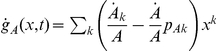
, this results in
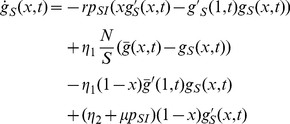
(5)

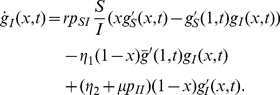
(6)Given that equations (3–4) show 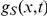 and its derivatives only for 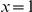 might suggest that the set of partial differential equations can be reduced to a set of ordinary differential equations including equations for the time evolution of the moments expressed by 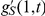 and 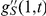. In fact, this leads to a hierarchy of equations for the time evolution of all higher order moments of 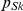.

With the equations given in Table 1 we are able to investigate the interplay between epidemic processes and transmission network topology. In particular, it is possible to follow the degree distribution within the subgroups of susceptible and infected individuals in terms of the probability generating functions 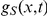 and 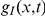. However, transmission networks do not only change their structure as a result of birth and death processes but also because of changes in contact partners. Analogous to [20], we consider swapping of contact partners at a rate 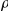 which affects the quantities 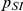 and 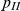 via additional terms 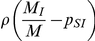 and 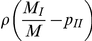, respectively.

### Implementation

Agent-based simulations were performed using NetLogo V4.0.4 [27], in which some code fragments from the model “Virus on Network” included in the software's model library were used [28]. Poisson networks were generated by assigning 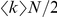 links between randomly chosen nodes; other random networks were generated based on their degree sequence [13], [29] (See Text S1 for details). The numerical solution to the partial differential equations was obtained using Mathematica V6.0.2 [30] with the function NDSolve and the numerical method of lines [31].

## Results

### Validation of the SID model

**Figure 1:**
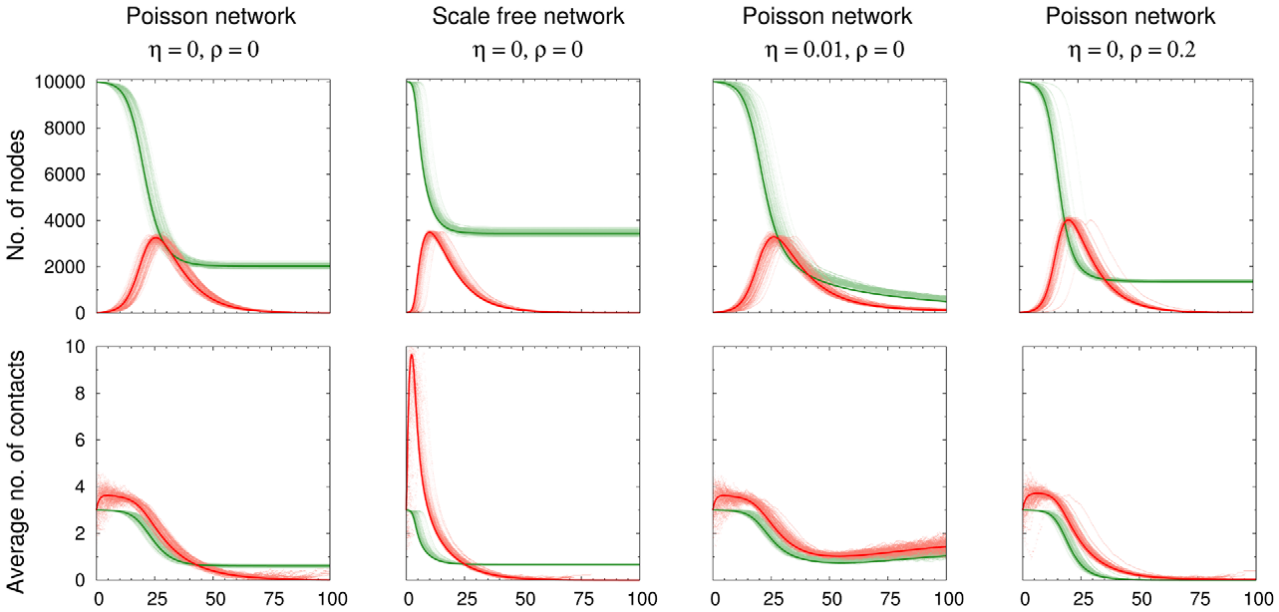
Evolution of the numbers of susceptible (green) and infected (red) individuals (top panel) as well as their average number of contacts per person (bottom panel). The dotted, light colored curves correspond to the result of 100 agent-based simulations; the solid lines are the results of the numerical solution of the set of partial differential equations (1–6), parameters are chosen analogous to [19], average number of contacts 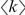 = 3, transmission rate of 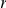 = 0.2, recovery rate of 
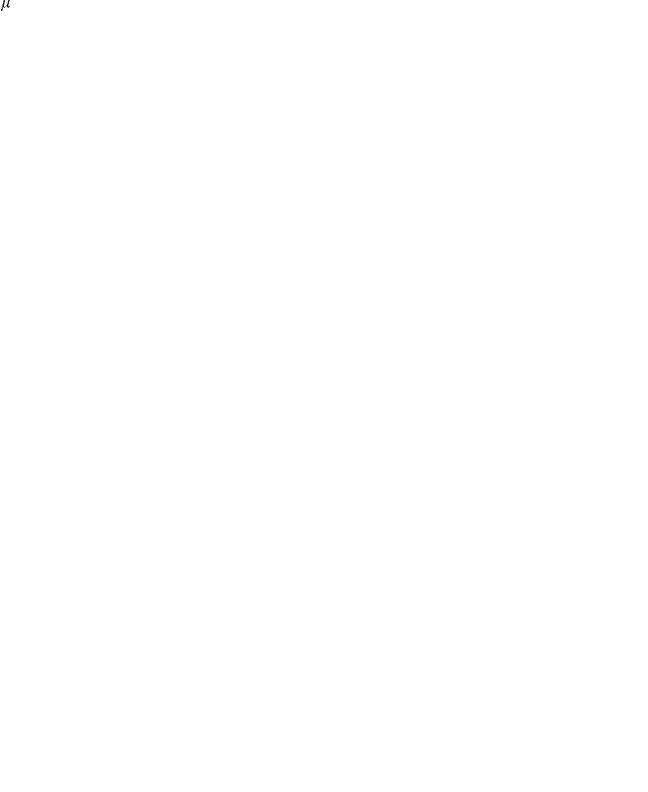
 = 0.1. Epidemics in networks with differences in the heterogeneity and transience of contacts are shown. The first column shows a static Poisson network with a degree distribution of 
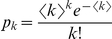
, as opposed to a network with a scale free degree distribution, 
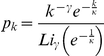
, in column 2 (same average degree). Column 3 corresponds to the network in column 1 with an additional demographic process (birth and death at a rate of 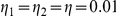). Column 4 corresponds to column 1 with the additional feature that contact partners change at a rate 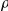 = 0.2. Epidemics are initiated with 10 infected individuals in an otherwise susceptible population (i.e. 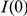 = 10, 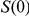 = 9990). Links between susceptible and infected hosts, as well as their contact behavior, are initially uncorrelated, i.e., 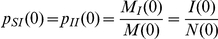.

The SID model allows for the study and analysis of epidemics using transmission networks with a broad range of topological features. In particular, any distribution of the number of infectious contacts per host can be implemented within the framework by providing the corresponding probability generating function as an input parameter. Fig. 1 compares the predictions derived from the set of partial differential equations (1–6) with the observations from agent based simulations for several exemplary topologies (See the section on Methods and Text S1 for details). Although equations (1–6) hold for the mean behavior in the limit of infinite network size, they are already a good approximation for the agent-based simulations with moderate population sizes and in particular reproduce well-characterized behaviors. Epidemics in heterogeneous networks spread faster but are more restricted (Fig. 1, column 1 vs. 2) [32]. Contrarily, transient contacts lead to a larger epidemic size when contacts are maintained sufficiently long to ensure transmission (Fig. 1, column 1 vs. 4). The re-growth in the average number of contacts per person (node) in the scenario with birth and death processes (Fig. 1, column 3) is reminiscent of re-emergent, or persistent, infections that can be observed in this context. Finally, the method can be naturally extended to epidemics of diseases with several infected stages before death (See Text S1).

### The SI_1_I_2_D model for HIV epidemics

To model HIV epidemics, we have to extend the SID model to take into account the heterogeneous infectious profile of an HIV infection. The course of the disease is characterized by a short, but highly infectious, period of primary infection. This is followed by a prolonged period of latent infection with a much lower infectiousness (sometimes referred to as an asymptomatic or chronic phase) [33] before the onset of AIDS as the final stage of the disease. Our focus will be on the primary infection, 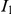, which ceases at a rate 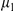 of 4.1 per year and is associated with a transmission rate 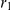 of 2.76 per year as opposed to the latent infection, 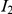, with a progression rate 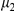 of 0.12 per year and a transmission rate 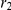 of 0.1 per year. We neglect the role of the final stage of disease in transmission with the assumption that health conditions prevent a further transmission of HIV. The evolution equations for 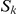, 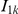 and 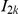 are determined analogously to the SID case to describe the numbers of individuals with 
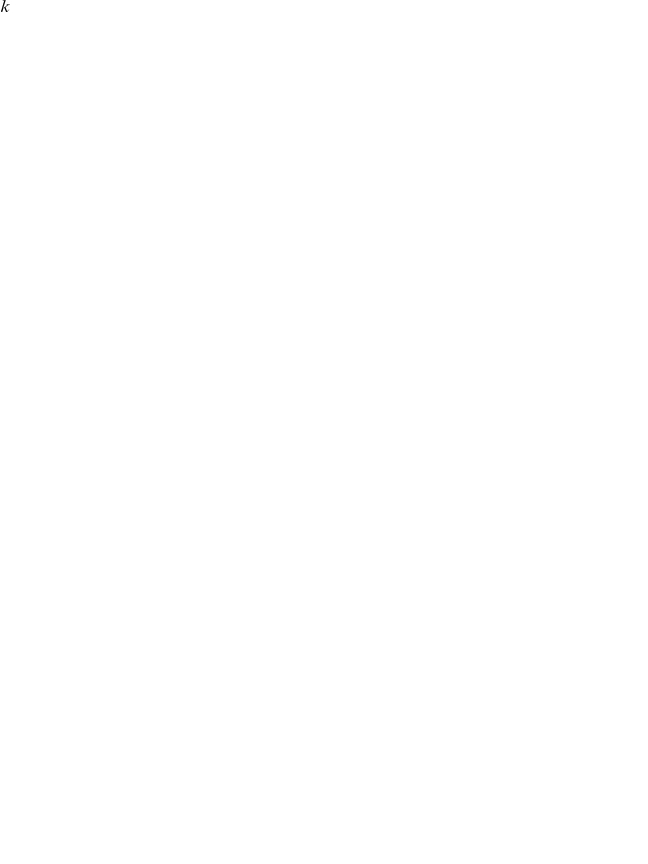
 contacts as being susceptible, primarily infected and latently infected. In addition, equations describing the contacts among individuals of different epidemic groups are derived (i.e., 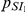, 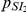, 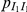, 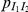 and 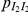). Finally, the set of equations is closed by a derivation of the probability generating functions 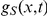, 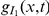 and 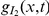 which describe the contact patterns and their temporal changes within each group (See Text S1 for details). The resulting equations allow for a much faster (and flexible) assessment of epidemic scenarios than agent-based simulations. Again, the structure of the equations emphasizes the mutual influence between the epidemic process and the underlying transmission network.

**Figure 2:**
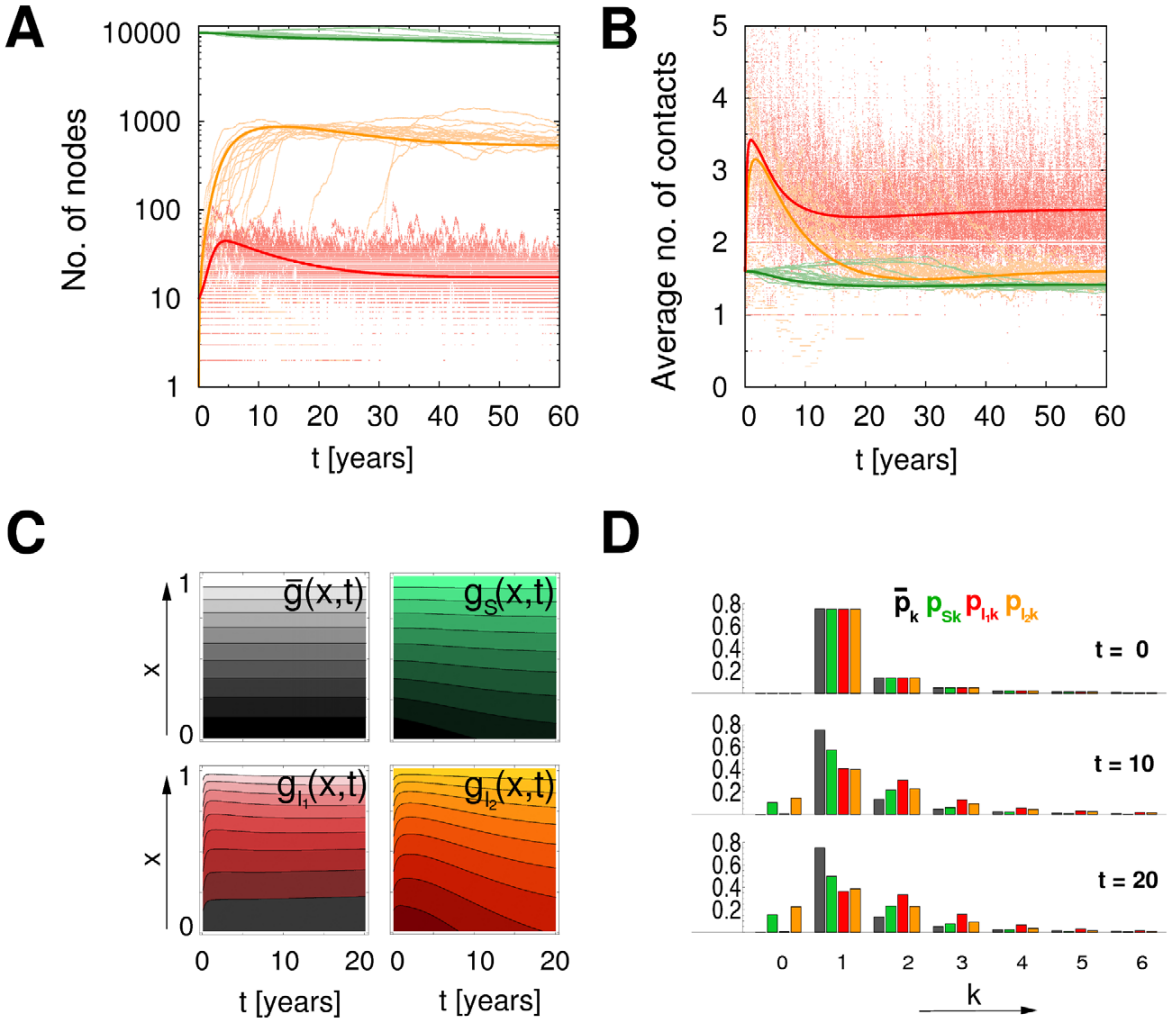
Evolution in the numbers of susceptible (green), primarily (red) and latently (orange) infected individuals as well as in their contact behavior. Evolution in the numbers of susceptible, primarily and latently infected individuals is shown in panel A, their average number of contacts per person during the epidemic is presented in panel B. The dotted, light colored curves correspond to the results of 20 agent-based simulations. The solid lines are the result of the numerical solution of the set of partial differential equations. A logarithmic scale was chosen to present the different orders of magnitude in the size of the epidemic subgroups. The epidemics take place on a scale free network with an average degree 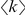 = 1.6 into which individuals are born at a rate of 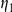 = 0.02 p.a. and die at a rate of 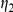 = 0.015 p.a.. The epidemic parameters are chosen in accordance with the infectious profile of HIV [33], i.e., transmission rates in the stages of primary and latent infection are 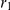 = 2.76 p.a. and 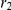 = 0.1 p.a., respectively, with rates of progression of 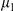 = 4.1 p.a. and 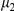 = 0.12 p.a.. Epidemics are initiated with 10 infected individuals in an otherwise susceptible population (i.e., 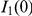 = 9, 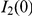 = 1, 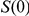 = 9990). Links between susceptible and infected hosts, as well as their contact behavior, are initially uncorrelated, i.e. 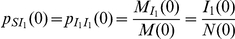, 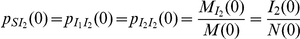. Panels C and D show the evolution of the probability generating functions and distributions in the number of contacts over 20 years of the epidemic for individuals newly entering the population (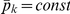, 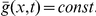) as well as in susceptible, primarily and latently infected individuals (
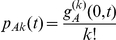
, 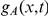, 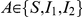). The contour plots of the PGFs interpolate from 0 (dark colors) to 1 (light colors) in steps of 0.1.

This is exemplarily illustrated in Fig. 2, which shows the spreading of HIV in a “synthetic” population with a scale free distribution in the number of potentially infectious contacts. Agent-based simulations and the numerical solution of the set of partial differential equations agree well and show how the epidemic saturates within a few decades with a few percent of latently infected individuals (as opposed to a few per mill of primarily infected individuals). During the epidemic expansion phase, the average number of contacts among infected individuals grows sharply while the average number of contacts among susceptible individuals decreases slightly. Individuals with more potentially infectious contacts are at a higher risk of infection and accumulate among primarily infected individuals. Their average number of contacts decreases during the following latent stage due to the increased mortality in their infected neighbors. This hierarchy in the average number of contacts from primarily infected through latently infected to susceptible individuals can still be observed during the saturation phase of the epidemic. The temporal evolution in the network topology can be studied in more detail from the probability generating functions 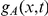 and the degree distributions 
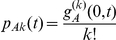
 (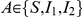) describing the contact behavior within the epidemic subgroups in Fig. 2 (panels C and D). Although the contact behavior of individuals newly entering the population is not time dependent and corresponds to the original distribution found before the onset of the epidemic (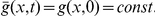), contact patterns change specifically in the epidemic subgroups. One can observe that the fraction of single individuals grows among susceptible individuals as it does among latently infected individuals in the saturation phase of the disease due to the increased mortality rate among their infected contacts. This loss in contacts is not observed in the short period of primary infection, which can be seen in the maintenance of the high average number of contacts in this subgroup (originally acquired due to the hazard of infection growing with the number of contacts). These features have been observed in actual HIV epidemics, for example in the Eighties' San Francisco MSM cohort [34]. Note that a persistent epidemic cannot be generally expected if new individuals enter the population with the current contact behavior instead of the initial contact behavior, i.e. with 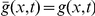 instead of 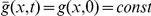. Because high-risk individuals have a higher risk of death due to the epidemic, this will successively lead to an introduction of individuals with lower risk behavior (lower average number of contacts) until eventually the network becomes sub-critical and the epidemic ceases [35].

### Transmission by stage of disease

The analysis has already taken into account that the course of an HIV infection is intimately related to the dynamics of its epidemic spreading. While the brief period of primary infection is associated with a largely increased infectiousness, a much lower infectiousness is observed during the prolonged period of latent infection (also referred to as asymptomatic or chronic infection), which grows again in the late stages of disease [33], [36]–[38]. Therefore, it cannot easily be determined, which phase of the disease results in most new infections, making this a topic of ongoing debate [39], [40]. Various studies on the contribution of the initial and latent stage to HIV incidence in different populations [41], [42] reveal that the contribution of either stage of the disease to HIV incidence is very context dependent. There is, however, agreement that primary infection becomes a more important epidemic driver when risk behavior increases (number of casual/concurrent contacts or partners), whereas the incidence obtained from latent infections becomes more important during the saturation phase of an epidemic (as opposed to its expansion phase), which is supported by modeling approaches [43], [44].

**Figure 3:**
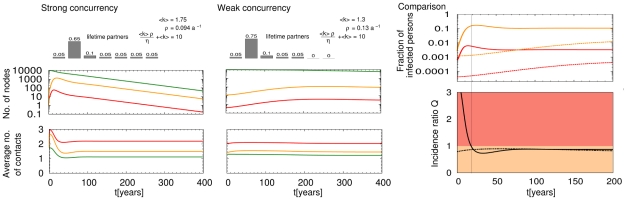
HIV epidemics in synthetic populations with weak and strong concurrency. Evolution in the numbers of susceptible (green), primarily (red) and latently (orange) infected individuals as well as their average number of contacts per person in the scenario of strong and weak concurrency are shown in the left and middle column. The logarithmic scale was chosen to present the different orders of magnitude in the size of the epidemic subgroups. The epidemics take place on random networks with the sketched distributions in the number of contacts (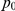 to 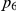 are shown, 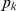 = 0 for 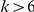) into which individuals are born and die at a rate of 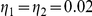 p.a.. The epidemic parameters were chosen in accordance with the infectious profile of HIV [33], i.e., transmission rates in the stages of primary and latent infection are 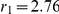 p.a. and 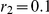 p.a., respectively, with rates of progression of 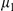 = 4.1 p.a. and 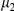 = 0.12 p.a.. The right column (comparison, top) shows the fraction of individuals in the primary (red) and latent (orange) stage of disease in the scenarios of strong concurrency (solid lines) and weak concurrency (dashed lines), for comparison. The final diagram shows the ratio, 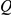, for both scenarios, i.e. the relative risk of infection acquired from primary over that from latent infections (strong concurrency - solid line, weak concurrency - dashed line). Epidemics are initiated as in Fig. 2, for better comparability the time axis was shifted to start both epidemics with the same number of latent cases after initial equilibration.

This question can certainly not be conclusively answered by our study due to a lack of adequate empirical data, but we can provide a tool that helps researchers to better understand under which circumstances infections dominantly originate from either primarily or latently infected individuals. Therefore, we do not aim to parameterize the model in the most realistic way but rather to investigate two possible scenarios that contain some general features found in epidemic networks of sexually transmitted HIV. There is generally a large heterogeneity in the numbers of sex partners [45]–[47], the frequency of partner change and the level of concurrency in partnerships [48]. Acknowledging that not only the number of infectious contacts but also their timing [49]–[51] is important for epidemic spreading, we investigate two scenarios that we depict as having weak and strong concurrency with parameters given in Fig. 3. Both scenarios assume an average number of 10 lifetime partners during a lifetime of 50 years (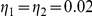 p.a., neglecting delays due to childhood/adolescence before sexual debut). However, in the scenario of weak concurrency, a lower average number of concurrent partners, 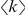, is exchanged for a higher partner change rate, 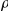, in comparison to the scenario of strong concurrency.

The numerical solution of the set of PDEs shown in Fig. 3 makes it immediately apparent that not only the number of partners but also the level of their concurrency have a profound impact on both the time scale and width of an epidemic's expansion. A much more severe epidemic is seen in the case of strong concurrency, which agrees with the earlier findings discussed above. This effect is still present if constant transmission rates are assumed throughout the course of disease (See Fig. 2 in Text S1). A common feature of both epidemics is the hierarchy in the average number of contacts for primarily, latently infected and susceptible individuals; this gives direction for targeted intervention strategies. To understand which stage of disease drives the epidemics, it is of particular interest to study the relative risk of infections derived from primary versus latent infections
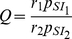
(7)which is shown in the bottom right panel of Fig. 3. While infections from the primary stage of disease dominate in the expansion phase of the epidemic in a scenario with strong concurrency in contacts, infections from the latent stage of disease dominate as soon as the epidemic matures. In the case of weak concurrency (being a closer approximation to serial monogamy) infections from the latent stage of disease dominate throughout the whole epidemic. This case study confirms that the question which stage of the disease drives HIV epidemics cannot be answered without in depth knowledge of the topology and dynamics of the underlying transmission network, as well as knowledge about the saturation stage of the epidemic.

## Discussion

A highly flexible mathematical framework has been introduced that allows for the investigation of the interplay between the topology and dynamics of transmission networks and emergent epidemics within a closed set of equations. The approach is focused on pathogens that lead to death of their hosts after some time of infection (potentially in several stages). HIV epidemics have been considered as an area of application in which the newly developed method helps to understand the complex interdependencies between the HIV epidemic profile, its transmission network, and the epidemic process. Several scenarios in synthetic populations have been investigated, showing that the transmission network is not a static support that shapes the epidemic but, on the contrary, is itself shaped by the epidemic. This becomes clear in the changing contact behavior of infected and susceptible individuals quantified either by degree distributions or probability generating functions. The mathematical framework further provides a capable tool to address the question of whether the group of primarily or of latently infected individuals is the main driver of HIV epidemics. The case studies emphasize that the answer depends both on the maturation stage of the epidemics and the structure of the relevant transmission network. These findings are relevant for the implementation of targeted intervention strategies (e.g., promotion of behavioral changes or vaccination programs if available), and are particularly relevant to the ongoing debate on public health policies [41], [42].

The capacity of the modeling approach has been illustrated by example applications. However, its real strength is the development of a framework that allows for a quantitative and systematic assessment of the interdependencies and feedback mechanisms between transmission network dynamics and the spread of an epidemic. In particular, the detailed tracing of contact behavior in all epidemic groups, which may also undergo a flexible demographic process, goes beyond earlier approaches. The method can naturally be extended to other settings with a more complex infectious profile or to epidemics with a classical SIR dynamic. This makes it useful for a very broad spectrum of epidemic scenarios, which may include improved modeling of SIR-like infections such as measles, rubella, pertussis or influenza. The broad applicability of the approach makes it worthwhile to consider further improvements to stretch its limits towards an increasingly realistic description of the epidemics we face day-to-day. The finite size agent-based simulations shown in Fig. 1 and Fig. 2 for validation already indicate that the current approach is designed for the limit of infinite population sizes. Although the mean behavior is well represented already for moderate population sizes the approach does not account for obvious fluctuations. Recent research has shown that stochastic fluctuations may have a strong influence on real world epidemic phenomena such as re-emergent epidemics [52], [53] which in combination with other recently developed techniques makes this an exciting direction of future research [54]. It is further assumed that contacts are made randomly [13] without taking into account any preferences or correlations influencing their establishment. Social networks usually show clustered communities [55] and some degree of assortativity, i.e., individuals tend to mix with their likes [10], [14], [56]. This often results in the generation of core groups that sustain and drive an epidemic. Ongoing research efforts [17], [18], [25], [57], [58] in this field give directions for an extended model, ideally in combination with a more realistic description of transient contacts. Moreover, demographic change with random assignment of new contacts results in increasingly homogeneous contact behavior of older individuals in the network (See Fig. 3–5 in Text S1). A straight-forward extension of the approach would be to study other modes of contact establishment, such as preferential attachment with respect to degree. This will allow for a study of more complex topological evolution and its consequences. Finally, it should be considered that the transmission network is not only shaped by the epidemic process but also by active behavioral changes, such as social distancing or vaccination [59]–[64].

In conclusion, we have presented a new mathematical framework that allows researchers to closely monitor both the epidemic process and its transmission network for general SIR-like infections in an computationally efficient manner. The current method allows for great flexibility accounting for variability in transmission network topology and dynamics, as well as pathogen specific features. Nonetheless, it will be important to assess the method's limitations in the field after parameterization with appropriate empirical data. An exciting challenge for future research is to further expand its limits.

## Supporting Information

Text S1 The supporting Text S1 contains an in-detail derivation of the set of partial differential equations for the SID and SI_1_I_2_D models. It further includes a discussion of local clustering of infected cases, of the impact of concurrent and transient contacts, some results on networks with node age, and detailed information about the agent-based simulations.
(0.70 MB PDF)
