# Supplementary material for: Untangling the Interplay between Epidemic Spread and Transmission Network Dynamics
Source: PLoS Comput Biol. 2010 Nov 18;6(11):e1000984. doi: 10.1371/journal.pcbi.1000984 (PMC2987842; doi:10.1371/journal.pcbi.1000984)
Supplement: Text S1 — The supporting Text S1 contains an in-detail derivation of the set of partial differential equations for the SID and SI1I2D models. It further includes a discussion of local clustering of infected cases, of the impact of concurrent and transient contacts, some results on networks with node age, and detailed information about the agent-based simulations. (0.70 MB PDF) [file pcbi.1000984.s001.pdf]

Supporting Text S1 for the manuscript “Untangling the interplay between epidemic spread and transmission network dynamics”

Christel Kamp*

August 19, 2010

General remarks

Throughout the following paragraphs we use the following notation:

$\dot{f}(x,t) = \frac{\partial}{\partial t} f(x,t)$	partial derivative of function f with respect to t
$f'(x,t) = \frac{\partial}{\partial x} f(x,t)$	partial derivative of function f with respect to x
A_k	number of individuals in group A with k contacts
$A = \sum_k A_k$	number of individuals in group A
$N_k = \sum_A A_k$	number of individuals with k contacts
$N = \sum_k N_k$	total number of individuals
$p_{Ak} = \frac{A_k}{A}$	probability for an individual in group A to have k contacts
$g_A(x,t) = \sum_k p_{Ak}(t) x^k$	probability generating function (PGF) of $p_{Ak}(t)$
$\langle k \rangle_A = g'_A(1,t)$	average number of contacts of A individuals
$p_k = \frac{N_k}{N}$	probability for an individual to have k contacts
$g(x,t) = \sum_k p_k(t) x^k = \sum_A \frac{A}{N} g_A(x,t)$	probability generating function (PGF) of $p_k(t)$
$\langle k \rangle = g'(1,t)$	average number of contacts of individuals
$\bar{p}_k$	probability for a new individual entering the population to have k contacts
$\bar{g}(x,t) = \sum_k \bar{p}_k(t) x^k$	probability generating function (PGF) of $\bar{p}_k(t)$
$M_A = \sum_k k A_k = A g'_A(1,t)$	number of links coming from A individuals
$M = \sum_A M_A$	number of links
M_{AB}	number of links coming from A individuals and pointing to B individuals
$p_{AB} = \frac{M_{AB}}{M_A}$	probability for a link starting from an A individual to point to an B individual

Note that A and B correspond to the stages passed through during an infection, e.g., S for susceptible, I for infected etc.. As long as no ambiguities arise, partial derivatives with respect to time/spatial variables are denoted by a dot/prime throughout the manuscript, otherwise standard notation is used.

*Paul-Ehrlich-Institut, Federal Institute for Vaccines and Biomedicines, Paul-Ehrlich-Straße 51-59, 63225 Langen, Germany, kamch@pei.de

Equations for the SID model

We consider an epidemic of a disease that is transmitted at a rate, r, and from which infected individuals die at a rate μ. The demographics of the background population are determined by a birth rate η_1 and a death rate η_2. This leads to the following equations for the evolution of the number of susceptible and infected individuals with k (infectious) contacts:

$$\begin{array}{lll}
\dot{S}_k = & -rp_{SI}kS_k & \text{new infections} \\
 & +\eta_1 N\bar{p}_k - \eta_2 S_k & \text{natural birth and death} \\
 & +\eta_1 \bar{g}'(1,t)(S_{k-1} - S_k) & \text{contacts made with new nodes} \\
 & -\eta_2(kS_k - (k+1)S_{k+1}) & \text{contacts lost from dying nodes} \\
 & -\mu p_{SI}(kS_k - (k+1)S_{k+1}) & \text{contacts lost from nodes dying from infection} \\
\dot{I}_k = & +rp_{SI}kS_k & \text{new infections} \\
 & -(\eta_2 + \mu)I_k & \text{death due to natural causes and disease} \\
 & +\eta_1 \bar{g}'(1,t)(I_{k-1} - I_k) & \text{contacts made with new nodes} \\
 & -\eta_2(kI_k - (k+1)I_{k+1}) & \text{contacts lost from dying nodes} \\
 & -\mu p_{II}(kI_k - (k+1)I_{k+1}) & \text{contacts lost from nodes dying from infection.}
\end{array}$$

In summary

$$\begin{aligned}
\dot{S}_k &= -rp_{SI}kS_k + \eta_1 N\bar{p}_k - \eta_2 S_k + \eta_1 \bar{g}'(1,t)(S_{k-1} - S_k) - (\eta_2 + \mu p_{SI})(kS_k - (k+1)S_{k+1}) \quad (1) \\
\dot{I}_k &= +rp_{SI}kS_k - (\eta_2 + \mu)I_k + \eta_1 \bar{g}'(1,t)(I_{k-1} - I_k) - (\eta_2 + \mu p_{II})(kI_k - (k+1)I_{k+1}). \quad (2)
\end{aligned}$$

Note that these equations imply that individuals who enter the population at a rate η_1 are susceptible and have k contacts with a probability of $\bar{p}_k$ by which they are randomly linked to individuals already present in the population. The following calculations rely on this random establishment of new contacts; however, other patterns of contact establishment, for example preferential attachment to nodes with respect to degree, could also naturally be implemented. Individuals dying from natural causes at a rate of η_2 are assumed to have an average number of contacts as found in the total population and do not show preferences to have contacted susceptible or infected individuals.

The terms in equations (1) and (2) which contain p_{SI} and p_{II} imply approximations which mediate the transition from more general pair models to individual based probability generating function approaches [1]. The number of susceptible individuals with k contacts decreases due to infection at a rate proportional to the transmission rate r and the number of links between them and infected individuals M_{S_kI} ($M_{S_kI} = [S_kI]$ in pair model notation, analogous for contacts lost from individuals dying from infection). Equations (1) and (2) assume that the probability for a link starting from a susceptible or infected node to point to an infected node is independent of the starting node's degree k, i.e.

$$-rM_{S_kI} \approx -rM_{SI}\frac{kS_k}{\sum_k kS_k} = -r\frac{M_{SI}}{M_S}kS_k = -rp_{SI}kS_k \quad (3)$$

$$\begin{aligned}
-\mu(M_{I_kI} - M_{I_{k+1}I}) &\approx -\mu M_{II}\left(\frac{kI_k}{\sum_k kI_k} - \frac{(k+1)I_{k+1}}{\sum_k kI_k}\right) = -\mu\frac{M_{II}}{M_I}(kI_k - (k+1)I_{k+1}) \\
&= -\mu p_{II}(kI_k - (k+1)I_{k+1}). \quad (4)
\end{aligned}$$

Therefore, p_{SI} and p_{II} are not explicitly dependent on the starting node's degree k, they however reflect different average contact behavior within the group of susceptible and infected hosts (cf. Fig. 1 of this document and of the main manuscript, for an in detail discussion of the connection between pair models with moment closure and individual based PGF models considering also performance issues cf. [1]).

Adding up the contributions of S_k and I_k for all k results in equations for the total number of susceptible and infected hosts:

$$\dot{S} = \eta_1 N - r p_{SI} M_S - \eta_2 S \tag{5}$$
$$\dot{I} = r p_{SI} M_S - (\eta_2 + \mu) I. \tag{6}$$

To close this set of equations we also need to derive equations for p_{SI} and p_{II}, as well as for the probability generating functions (PGF) $g_S(x,t)$ and $g_I(x,t)$.

We start with the probabilities for a link starting from a susceptible or infected individual to point to an infected individual, p_{SI} and p_{II}, respectively. This takes into account that there are different probabilities for susceptible and infected individuals to have infected contacts (cf. Fig. 1). Following the argument in [2] we write the evolution equations for $p_{SI} = M_{SI}/M_S$ and $p_{II} = M_{II}/M_I$ in terms of links among susceptible and infected hosts:

$$\dot{p}_{SI} = \frac{\dot{M}_{SI}}{M_S} - \frac{\dot{M}_S}{M_S} p_{SI} \tag{7}$$
$$\dot{p}_{II} = \frac{\dot{M}_{II}}{M_I} - \frac{\dot{M}_I}{M_I} p_{II} \tag{8}$$

for which expressions are derived in the following paragraph. From the definition of M_S and M_I equations for their temporal evolution can be derived as

$$\dot{M}_S = \sum_k k \dot{S}_k \tag{9}$$
$$\dot{M}_I = \sum_k k \dot{I}_k. \tag{10}$$

After substitution from equations (1-2) this results in

$$\dot{M}_S = -r p_{SI} S (g_S''(1,t) + g_S'(1,t)) + \eta_1 \bar{g}'(1,t)(N+S) - (2\eta_2 + \mu p_{SI}) M_S \tag{11}$$
$$\dot{M}_I = = r p_{SI} S (g_S''(1,t) + g_S'(1,t)) + \eta_1 \bar{g}'(1,t) I - (2\eta_2 + \mu + \mu p_{II}) M_I. \tag{12}$$

The temporal change in M_S and M_I is affected by the epidemic process (first contribution), contacts made from new nodes as well as contacts of new nodes for M_S (second contribution) and by dying nodes and their bi-directional contacts (third contribution).

Following arguments analogous to those in [2] one can show that the probability generating function of the joint probability to find a susceptible node with $k-i$ links to susceptible nodes and i links to infected nodes is generated by $g_S(x_S p_{SS} + x_I p_{SI}) = g_S(x_S(1-p_{SI}) + x_I p_{SI})$. This relies on the assumption that the number of contacts from susceptible hosts to susceptible and infected hosts is binomially distributed with probabilities p_{SI} and $p_{SS} = 1 - p_{SI}$. The probability for a susceptible node that was reached from an infected node (i.e., chosen with probability proportional to the number of contacts pointing to infected nodes i) to have $(i-1)$ contacts to infected nodes and $k-i$ contacts to susceptible nodes is generated by $\frac{g_S'(x_S(1-p_{SI})+x_I p_{SI})}{g_S'(1)}$. Therefore, the average excess degrees of a susceptible node that was reached from an infected node to susceptible or infected nodes are

$$\delta_{SI}(S) = \frac{\partial}{\partial x_S} \frac{g_S'(x_S(1-p_{SI}) + x_I p_{SI})}{g_S'(1)}_{|x_S = x_I = 1} = (1-p_{SI}) \frac{g_S''(1,t)}{g_S'(1,t)} \tag{13}$$
$$\delta_{SI}(I) = \frac{\partial}{\partial x_I} \frac{g_S'(x_S(1-p_{SI}) + x_I p_{SI})}{g_S'(1)}_{|x_S = x_I = 1} = p_{SI} \frac{g_S''(1,t)}{g_S'(1,t)}. \tag{14}$$

Bookkeeping of the changes in the numbers of links among susceptible and infected hosts due to the epidemic and demographic process results in:

$\dot{M}_{SI} =$	$-rp_{SI}M_S(\delta_{SI}(I) - \delta_{SI}(S))$	change due to epidemic spread: change in the number of susceptible nodes $rp_{SI}M_S$ due to the epidemic times their average excess contacts to susceptible and infected nodes
	$-rM_{SI}$	discount for link along which the infection spread
	$-\mu M_{SI}$	link loss due to disease progression
	$-2\eta_2 M_{SI}$	link loss due to natural death
	$+\eta_1 \bar{g}'(1,t)I$	link addition due to birth
$\dot{M}_{II} =$	$2rp_{SI}M_S\delta_{SI}(I)$	change due to epidemic spread: change in the number of susceptible nodes $rp_{SI}M_S$ due to the epidemic times their average excess contacts to infected nodes (bi-directional)
	$+2rM_{SI}$	add link along which infection spread (bi-directional)
	$-2\mu M_{II}$	link loss due to disease progression
	$-2\eta_2 M_{II}$	link loss due to natural death.

In summary this results in

$$\begin{aligned}
\dot{M}_{SI} &= -rp_{SI}M_S(\delta_{SI}(I) - \delta_{SI}(S)) - (r+\mu)M_{SI} - 2\eta_2 M_{SI} + \eta_1\bar{g}'(1,t)I &(15)\\
&= -r(2p_{SI}-1)p_{SI}M_S\frac{g_S''(1,t)}{g_S'(1,t)} - (r+\mu+2\eta_2)M_{SI} + \eta_1\bar{g}'(1,t)I &(16)\\
\dot{M}_{II} &= 2rp_{SI}M_S\delta_{SI}(I) + 2rM_{SI} - 2\mu M_{II} - 2\eta_2 M_{II} &(17)\\
&= 2rp_{SI}^2M_S\frac{g_S''(1,t)}{g_S'(1,t)} + 2rM_{SI} - 2(\mu+\eta_2)M_{II}, &(18)
\end{aligned}$$

which finally leads to

$$\begin{aligned}
\dot{p}_{SI} &= rp_{SI}(1-p_{SI})\frac{g_S''(1,t)}{g_S'(1,t)} - (r+\mu)p_{SI}(1-p_{SI}) + \eta_1\frac{\bar{g}'(1,t)}{M_S}(I-(N+S)p_{SI}) &(19)\\
\dot{p}_{II} &= r\frac{M_S}{M_I}p_{SI}(2p_{SI}-p_{II})\frac{g_S''(1,t)}{g_S'(1,t)} - r\frac{M_S}{M_I}p_{SI}p_{II} + 2r\frac{M_S}{M_I}p_{SI} - \mu(1-p_{II})p_{II} + \eta_1\frac{\bar{g}'(1,t)}{M_I}Ip_{II}. &(20)
\end{aligned}$$

p_{SI} and p_{II} are not explicitly dependent on the node degree k. However, they describe the probabilities for a link that starts from a susceptible or infected node to point to an infected node. Infected nodes have, on average, a higher degree than susceptible nodes as long as the epidemic does not die out (cf. Fig. 1 of the main manuscript). To close the set of equations we also need to derive equations for the probability generating functions (PGF) $g_S(x,t)$ and $g_I(x,t)$ which correspond to the probabilities to find individuals with k contacts among the susceptible and infected hosts, respectively, i.e., p_{Sk} and p_{Ik}. From the definitions of the PGFs we obtain

$$\begin{aligned}
\dot{g}_S(x,t) &= \sum_k \left(\frac{\dot{S}_k}{S} - \frac{\dot{S}}{S}p_{Sk}\right)x^k &(21)\\
\dot{g}_I(x,t) &= \sum_k \left(\frac{\dot{I}_k}{I} - \frac{\dot{I}}{I}p_{Ik}\right)x^k, &(22)
\end{aligned}$$

which results in

$$\begin{aligned}\dot{g}_S(x,t) &= -rp_{SI}(xg'_S(x,t) - g'_S(1,t)g_S(x,t)) + \eta_1\frac{N}{S}(\bar{g}(x,t) - g_S(x,t)) \\ &\quad -\eta_1(1-x)\bar{g}'(1,t)g_S(x,t) + (\eta_2 + \mu p_{SI})(1-x)g'_S(x,t) \qquad (23)\\ \dot{g}_I(x,t) &= rp_{SI}\frac{S}{I}(xg'_S(x,t) - g'_S(1,t)g_I(x,t)) \\ &\quad -\eta_1(1-x)\bar{g}'(1,t)g_I(x,t) + (\eta_2 + \mu p_{II})(1-x)g'_I(x,t). \qquad (24)\end{aligned}$$

The probability generating function $g(x,t)$ of the total population's degree distribution p_k reads

$$\begin{aligned}\dot{g}(x,t) = {}& \mu\frac{I}{N}(g(x,t) - g_I(x,t)) + \eta_1(\bar{g}(x,t) - g(x,t)) - \eta_1(1-x)\bar{g}'(1,t)g(x,t) \\ &+\eta_2(1-x)g'(x,t) + \mu(1-x)(\frac{S}{N}p_{SI}g'_S(x,t) + \frac{I}{N}p_{II}g'_I(x,t)). \qquad (25)\end{aligned}$$

Noticing that equations (19) and (20) contain $g_S(x,t)$ and its derivatives only for $x = 1$ suggests that the set of partial differential equations can be reduced to a set of ordinary differential equations including the moments of p_{Sk} expressed by $g'_S(1,t) = \langle k\rangle_S$ and $g''_S(1,t) = \langle k^2\rangle_S - \langle k\rangle_S$. The mth partial derivative of $g(x,t)$ with respect to x at $x = 1$ and its derivative with respect to t can be written as

$$g_S^{(m)}(1,t) = \sum_k k(k-1)...(k-m+1)p_{Sk} = \sum_k k(k-1)...(k-m+1)\frac{S_k}{S} \qquad (26)$$

$$\dot{g}_S^{(m)}(1,t) = \sum_k k(k-1)...(k-m+1)\dot{p}_{Sk} = \sum_k k(k-1)...(k-m+1)\left(\frac{\dot{S}_k}{S} - \frac{\dot{S}}{S}p_{Sk}\right) \qquad (27)$$

in which $\dot{p}_{Sk}$ can be derived by substitution from equations (1) and (5) to be

$$\begin{aligned}\dot{p}_{Sk} &= \left(\frac{\dot{S}_k}{S} - \frac{\dot{S}}{S}p_{Sk}\right) \qquad (28)\\ &= -rp_{SI}(k - \langle k\rangle_S)p_{Sk} + \eta_1\frac{N}{S}(\bar{p}_k - p_{Sk}) + \eta_1\langle k\rangle_S(p_{Sk-1} - p_{Sk}) - (\eta_2 + \mu p_{SI})(kp_{Sk} - (k+1)p_{Sk+1}).\end{aligned}$$

Due to the first term (i.e. $-rp_{SI}kp_{Sk}$) in equation (28) equations for the temporal evolution of $g^{(m)}(1,t)$ (or the mth moment of p_{Sk}) will depend on $g^{(m+1)}(1,t)$, i.e. the next higher order moment of p_{Sk}. This results in a hierarchy of ordinary differential equations for $g^{(m)}(1,t)$ (or the moments of p_{Sk}, respectively).

Complementarily to the figures shown in the main manuscript, Fig. 1 shows the time evolution in the probabilities p_{SI} and p_{II} in comparison with the values expected under random mixing M_I/M. The deviations are a clear indicator of local clustering of infected cases.

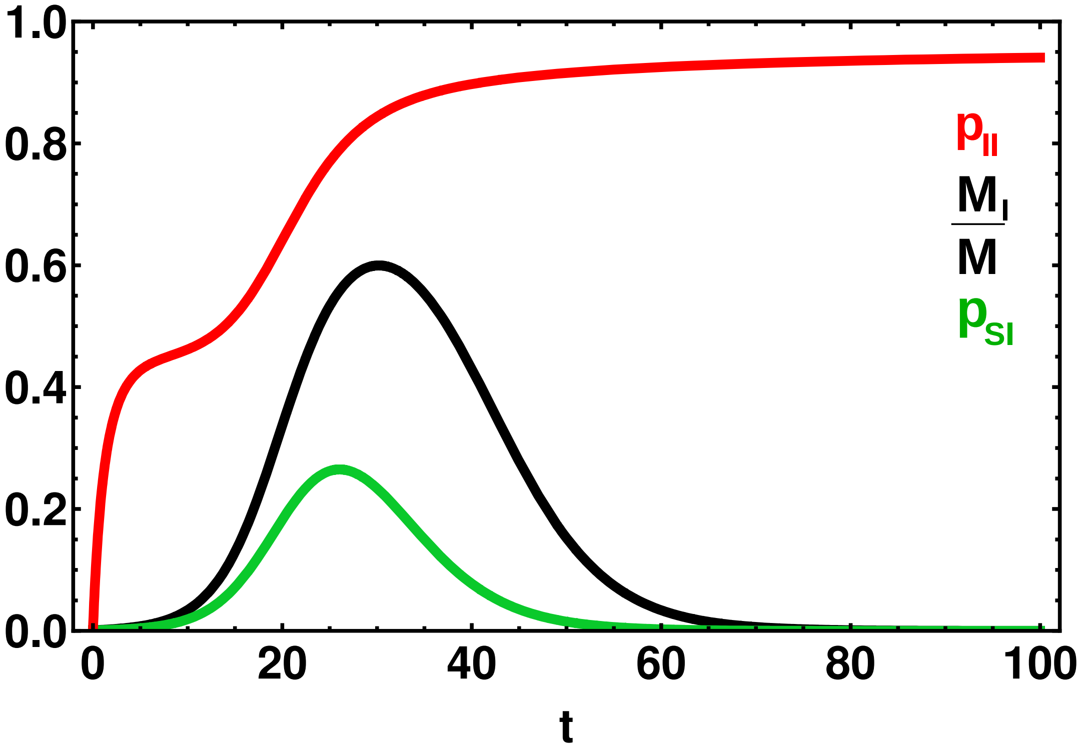

Figure 1: Evolution in the probabilities of contacts from susceptible or infected hosts to be made with infected hosts p_{SI} (green) and p_{II} (red) in comparison with the total fraction of contacts with infected hosts M_I/M (black). Parameters were chosen analogous to Fig. 1 in the main manuscript, i.e., average number of contacts $\langle k \rangle = 3$, $p_k = \frac{\langle k \rangle^k e^{-\langle k \rangle}}{k!}$, transmission rate of $r = 0.2$ and a recovery rate of $\mu = 0.1$ (initial conditions were $p_{SI}(0) = p_{II}(0) = M_I(0)/M(0) = I(0)/N = 0.001$). Note that infected hosts are more likely to link to infected hosts than expected by random mixing (i.e., M_I/M), whereas susceptible hosts are less likely to do so. This is a consequence of local clustering of infected cases.

Equations for the SI$_1$I$_2$D model

We extended the equations of the SID model to accommodate two infected stages, I_1 and I_2, with transmission rates of r_1 and r_2, which cease at rates μ_1 and μ_2, respectively. The equations for the number of individuals with k contacts who are in the classes S, I_1 or I_2 read accordingly:

$$\begin{aligned} \dot{S}_k &= -(r_1 p_{SI_1} + r_2 p_{SI_2}) k S_k + \eta_1 N \bar{p}_k - \eta_2 S_k + \eta_1 \bar{g}'(1,t)(S_{k-1} - S_k) \\ &\quad -(\eta_2 + \mu_2 p_{SI_2})(k S_k - (k+1) S_{k+1}) \qquad (29) \\ \dot{I}_{1k} &= +(r_1 p_{SI_1} + r_2 p_{SI_2}) k S_k - (\eta_2 + \mu_1) I_{1k} + \eta_1 \bar{g}'(1,t)(I_{1k-1} - I_{1k}) \\ &\quad -(\eta_2 + \mu_2 p_{I_1 I_2})(k I_{1k} - (k+1) I_{1k+1}) \qquad (30) \\ \dot{I}_{2k} &= \mu_1 I_{1k} - (\eta_2 + \mu_2) I_{2k} + \eta_1 \bar{g}'(1,t)(I_{2k-1} - I_{2k}) \\ &\quad -(\eta_2 + \mu_2 p_{I_2 I_2})(k I_{2k} - (k+1) I_{2k+1}). \qquad (31) \end{aligned}$$

In terms of the total number of susceptible and infected individuals of either stage, the equations read (after adding up the contributions for all k)

$$\begin{aligned} \dot{S} &= \eta_1 N - (r_1 p_{SI_1} + r_2 p_{SI_2}) M_S - \eta_2 S && (32) \\ \dot{I}_1 &= (r_1 p_{SI_1} + r_2 p_{SI_2}) M_S - (\eta_2 + \mu_1) I_1 && (33) \\ \dot{I}_2 &= \mu_1 I_1 - (\eta_2 + \mu_2) I_2. && (34) \end{aligned}$$

To derive the probabilities p_{AB} for links from A to point to B we again use

$$\dot{p}_{AB} = \frac{\dot{M}_{AB}}{M_A} - \frac{\dot{M}_A}{M_A} p_{AB} \tag{35}$$

with $A, B \in \{S, I_1, I_2\}$. Analogous to [2] and to the case of the SID model, we derive that the average excess degree of a susceptible node that was reached from an infected node (either of I_1 or I_2 type) to susceptible or infected nodes are $\delta_{SI}(I_1) = p_{SI_1} \frac{g_S''(1,t)}{g_S'(1,t)}$, $\delta_{SI}(I_2) = p_{SI_2} \frac{g_S''(1,t)}{g_S'(1,t)}$ or $\delta_{SI}(S) = (1 - p_{SI_1} - p_{SI_2}) \frac{g_S''(1,t)}{g_S'(1,t)}$, respectively. This relies on the assumption that the number of contacts from susceptible hosts to susceptible, primarily and latently infected hosts is multinomially distributed with probabilities p_{SI_1}, p_{SI_2} and $p_{SS} = 1 - p_{SI_1} - p_{SI_2}$. With this, we can write down the contributions that change the numbers of contacts among susceptible and infected hosts of either stage (i.e., M_A, M_{AB}, $A, B \in \{S, I_1, I_2\}$) and summarize as

$$\begin{aligned} \dot{M}_S &= -(r_1 p_{SI_1} + r_2 p_{SI_2}) S (g_S''(1,t) + g_S'(1,t)) + \eta_1 \bar{g}'(1,t)(N + S) - (2\eta_2 + \mu_2 p_{SI_2}) M_S && (36) \\ \dot{M}_{I_1} &= (r_1 p_{SI_1} + r_2 p_{SI_2}) S (g_S''(1,t) + g_S'(1,t)) + \eta_1 \bar{g}'(1,t) I_1 - (2\eta_2 + \mu_1 + \mu_2 p_{I_1 I_2}) M_{I_1} && (37) \\ \dot{M}_{I_2} &= \mu_1 M_{I_1} + \eta_1 \bar{g}'(1,t) I_2 - (2\eta_2 + \mu_2 + \mu_2 p_{I_2 I_2}) M_{I_2} && (38) \end{aligned}$$

$$\begin{aligned} \dot{M}_{SI_1} &= -(r_1 p_{SI_1} + r_2 p_{SI_2}) M_S (\delta_{SI}(I_1) - \delta_{SI}(S)) - (r_1 + \mu_1) M_{SI_1} - 2\eta_2 M_{SI_1} + \eta_1 \bar{g}'(1,t) I_1 && (39) \\ &= -(r_1 p_{SI_1} + r_2 p_{SI_2})(2 p_{SI_1} + p_{SI_2} - 1) M_S \frac{g_S''(1,t)}{g_S'(1,t)} - (r_1 + \mu_1 + 2\eta_2) M_{SI_1} + \eta_1 \bar{g}'(1,t) I_1 && (40) \\ \dot{M}_{SI_2} &= -(r_1 p_{SI_1} + r_2 p_{SI_2}) M_S \delta_{SI}(I_2) - (r_2 + \mu_2) M_{SI_2} + \mu_1 M_{SI_1} - 2\eta_2 M_{SI_2} + \eta_1 \bar{g}'(1,t) I_2 && (41) \\ &= -(r_1 p_{SI_1} + r_2 p_{SI_2}) p_{SI_2} M_S \frac{g_S''(1,t)}{g_S'(1,t)} + \mu_1 M_{SI_1} - (r_2 + \mu_2 + 2\eta_2) M_{SI_2} + \eta_1 \bar{g}'(1,t) I_2 && (42) \\ \dot{M}_{I_1 I_1} &= 2(r_1 p_{SI_1} + r_2 p_{SI_2}) M_S \delta_{SI}(I_1) + 2 r_1 M_{SI_1} - 2\mu_1 M_{I_1 I_1} - 2\eta_2 M_{I_1 I_1} && (43) \\ &= 2(r_1 p_{SI_1} + r_2 p_{SI_2}) p_{SI_1} M_S \frac{g_S''(1,t)}{g_S'(1,t)} + 2 r_1 M_{SI_1} - 2(\mu_1 + \eta_2) M_{I_1 I_1} && (44) \end{aligned}$$

$$\begin{aligned} \dot{M}_{I_1 I_2} &= (r_1 p_{SI_1} + r_2 p_{SI_2}) M_S \delta_{SI}(I_2) + r_2 M_{SI_2} - (\mu_1 + \mu_2) M_{I_1 I_2} + \mu_1 M_{I_1 I_1} - 2\eta_2 M_{I_1 I_2} && (45) \\ &= (r_1 p_{SI_1} + r_2 p_{SI_2}) p_{SI_2} M_S \frac{g_S''(1,t)}{g_S'(1,t)} + r_2 M_{SI_2} + \mu_1 M_{I_1 I_1} - (\mu_1 + \mu_2 + 2\eta_2) M_{I_1 I_2} && (46) \\ \dot{M}_{I_2 I_2} &= 2\mu_1 M_{I_1 I_2} - 2(\mu_2 + \eta_2) M_{I_2 I_2}. && (47) \end{aligned}$$

From this, the equations for $p_{SI_1}, p_{SI_2}, p_{I_1I_1}, p_{I_1I_2}$ and $p_{I_2I_2}$ can be derived to be

$$\begin{aligned}
\dot{p}_{SI_1} &= (r_1 p_{SI_1} + r_2 p_{SI_2})(1 - p_{SI_1} - p_{SI_2})\frac{g''_S(1,t)}{g'_S(1,t)} + (r_1 p_{SI_1} + r_2 p_{SI_2})p_{SI_1} \\
&\quad -(r_1 + \mu_1 - \mu_2 p_{SI_2})p_{SI_1} + \eta_1 \frac{\bar{g}'(1,t)}{M_S}(I_1 - (N+S)p_{SI_1}) \qquad (48) \\
\dot{p}_{SI_2} &= (r_1 p_{SI_1} + r_2 p_{SI_2})p_{SI_2} + \mu_1 p_{SI_1} - (r_2 + \mu_2 - \mu_2 p_{SI_2})p_{SI_2} + \eta_1 \frac{\bar{g}'(1,t)}{M_S}(I_2 - (N+S)p_{SI_2}) \qquad (49) \\
\dot{p}_{I_1I_1} &= (r_1 p_{SI_1} + r_2 p_{SI_2})(2p_{SI_1} - p_{I_1I_1})\frac{M_S}{M_{I_1}}\frac{g''_S(1,t)}{g'_S(1,t)} - (r_1 p_{SI_1} + r_2 p_{SI_2})\frac{M_S}{M_{I_1}}p_{I_1I_1} + 2r_1\frac{M_S}{M_{I_1}}p_{SI_1} \\
&\quad -(\mu_1 - \mu_2 p_{I_1I_2})p_{I_1I_1} - \eta_1\frac{\bar{g}'(1,t)}{g'_{I_1}(1,t)}p_{I_1I_1} \qquad (50) \\
\dot{p}_{I_1I_2} &= (r_1 p_{SI_1} + r_2 p_{SI_2})(p_{SI_2} - p_{I_1I_2})\frac{M_S}{M_{I_1}}\frac{g''_S(1,t)}{g'_S(1,t)} - (r_1 p_{SI_1} + r_2 p_{SI_2})\frac{M_S}{M_{I_1}}p_{I_1I_2} + r_2\frac{M_S}{M_{I_1}}p_{SI_2} \\
&\quad +\mu_1 p_{I_1I_1} - \mu_2(1 - p_{I_1I_2})p_{I_1I_2} - \eta_1\frac{\bar{g}'(1,t)}{g'_{I_1}(1,t)}p_{I_1I_2} \qquad (51) \\
\dot{p}_{I_2I_2} &= \mu_1\frac{M_{I_1}}{M_{I_2}}(2p_{I_1I_2} - p_{I_2I_2}) - \mu_2(1 - p_{I_2I_2})p_{I_2I_2} - \eta_1\frac{\bar{g}'(1,t)}{g'_{I_2}(1,t)}p_{I_2I_2}. \qquad (52)
\end{aligned}$$

Again, to close the set of equations, we need to derive the probability generating functions

$$\dot{g}_A(x,t) = \sum_k \left(\frac{\dot{A}_k}{A} - \frac{\dot{A}}{A}p_{Ak}\right)x^k \text{ with } A \in \{S, I_1, I_2\}, \qquad (53)$$

to conclude with

$$\begin{aligned}
\dot{g}_S(x,t) &= \frac{(r_1 p_{SI_1} + r_2 p_{SI_2})}{S}(M_S g_S(x,t) - xSg'_S(x,t)) + \eta_1\frac{N}{S}(\bar{g}(x,t) - g_S(x,t)) \\
&\quad -\eta_1(1-x)\bar{g}'(1,t)g_S(x,t) + (\eta_2 + \mu_2 p_{SI_2})(1-x)g'_S(x,t) \qquad (54) \\
\dot{g}_{I_1}(x,t) &= -\frac{(r_1 p_{SI_1} + r_2 p_{SI_2})}{I_1}(M_S g_{I_1}(x,t) - xSg'_S(x,t)) \\
&\quad -\eta_1(1-x)\bar{g}'(1,t)g_{I_1}(x,t) + (\eta_2 + \mu_2 p_{I_1I_2})(1-x)g'_{I_1}(x,t) \qquad (55) \\
\dot{g}_{I_2}(x,t) &= \mu_1\frac{I_1}{I_2}(g_{I_1}(x,t) - g_{I_2}(x,t)) \\
&\quad -\eta_1(1-x)\bar{g}'(1,t)g_{I_2}(x,t) + (\eta_2 + \mu_2 p_{I_2I_2})(1-x)g'_{I_2}(x,t). \qquad (56)
\end{aligned}$$

Note that the global quantities can be easily derived from these equations

$$\begin{aligned}
\dot{N}_k &= \eta_1 N\bar{p}_k - \eta_2 N_k - \mu_2 I_{2k} + \eta_1\bar{g}'(1,t)(N_{k-1} - N_k) + \eta_2(kN_k - (k+1)N_{k+1}) \\
&\quad -\mu_2(p_{SI_2}(kS_k - (k+1)S_{k+1}) + p_{I_1I_2}(kI_{1k} - (k+1)I_{1k+1}) + p_{I_2I_2}(kI_{2k} - (k+1)I_{2k+1})) \qquad (57) \\
\dot{N} &= (\eta_1 - \eta_2)N - \mu_2 I_2 \qquad (58) \\
\dot{M} &= 2\eta_1\bar{g}'(1,t)N - 2\eta_2 g'(1,t)N - 2\mu_2 M_{I_2} \qquad (59) \\
\dot{g}(x,t) &= \mu_2\frac{I_2}{N}(g(x,t) - g_{I_2}(x,t)) + \eta_1(\bar{g}(x,t) - g(x,t)) - \eta_1(1-x)\bar{g}'(1,t)g(x,t) \\
&\quad +\eta_2(1-x)g'(x,t) + \mu_2(1-x)(\frac{S}{N}p_{SI_2}g'_S(x,t) + \frac{I_1}{N}p_{I_1I_2}g'_{I_1}(x,t) + \frac{I_2}{N}p_{I_2I_2}g'_{I_2}(x,t)). \qquad (60)
\end{aligned}$$

Transient contacts

Transient contacts are considered analogous to [3] by swapping of partners at a rate of ρ. That means that the identity of each contact of a node changes at a rate of ρ, i.e., highly connected nodes are more likely to establish new contacts. Transient contacts affect the probabilities for links originating from A nodes to point to B nodes p_{AB} via an additional term

$$-\rho p_{AB}\left(1-\frac{M_B}{M}\right)+\rho(1-p_{AB})\frac{M_B}{M}=\rho\left(\frac{M_B}{M}-p_{AB}\right) \tag{61}$$

in the differential equations with $A, B \in \{S, I, I_1, I_2\}$. An example of the impact of transient contacts on epidemics is shown for the SID case in Fig. 2.

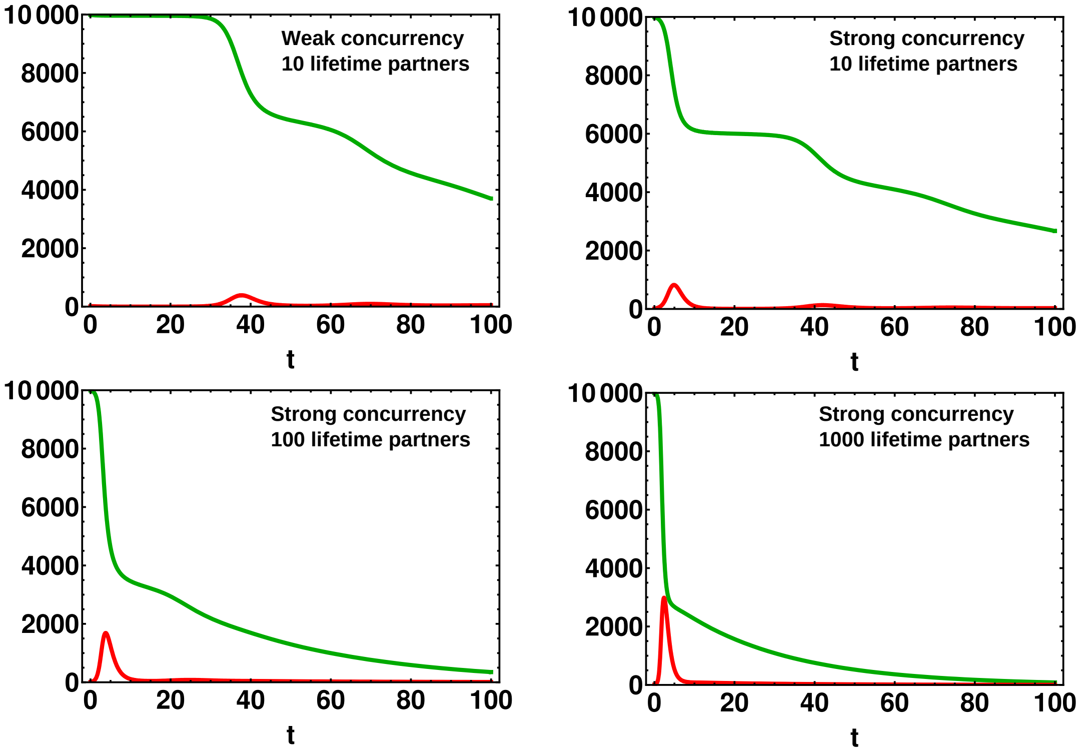

Figure 2: The impact of various levels of concurrency and transience in contact behavior is shown for epidemics with a transmission rate of $r = 2$, a progression rate of $\mu = 1$ and birth and death rate of $\eta = 0.02$. The scenarios of weak and strong concurrency refer to the degree distributions as shown in Fig. 3 of the main manuscript, i.e., with $\langle k\rangle = 1.3$ and $\langle k\rangle = 1.75$, respectively. Contacts change at a rate ρ so that the number of lifetime partners is $\frac{\langle k\rangle\rho}{\eta} + \langle k\rangle = 10$, 100 and 1000.

Networks with node age

To distinguish between the topological changes introduced by demographic and epidemic processes, we focus on network evolution under a demographic process without an epidemic. In this case, the equation (25) reduces to

$$\dot{g}(x,t) \;=\; \eta_1(\bar{g}(x,t) - g(x,t)) - \eta_1(1-x)\bar{g}'(1,t)g(x,t) + \eta_2(1-x)g'(x,t), \tag{62}$$

which can equivalently be written as

$$\frac{\partial}{\partial t}g(x,t) \;=\; \eta_1(\bar{g}(x,t) - g(x,t)) - \eta_1(1-x)\frac{\partial}{\partial x}\bar{g}(1,t)g(x,t) + \eta_2(1-x)\frac{\partial}{\partial x}g(x,t). \tag{63}$$

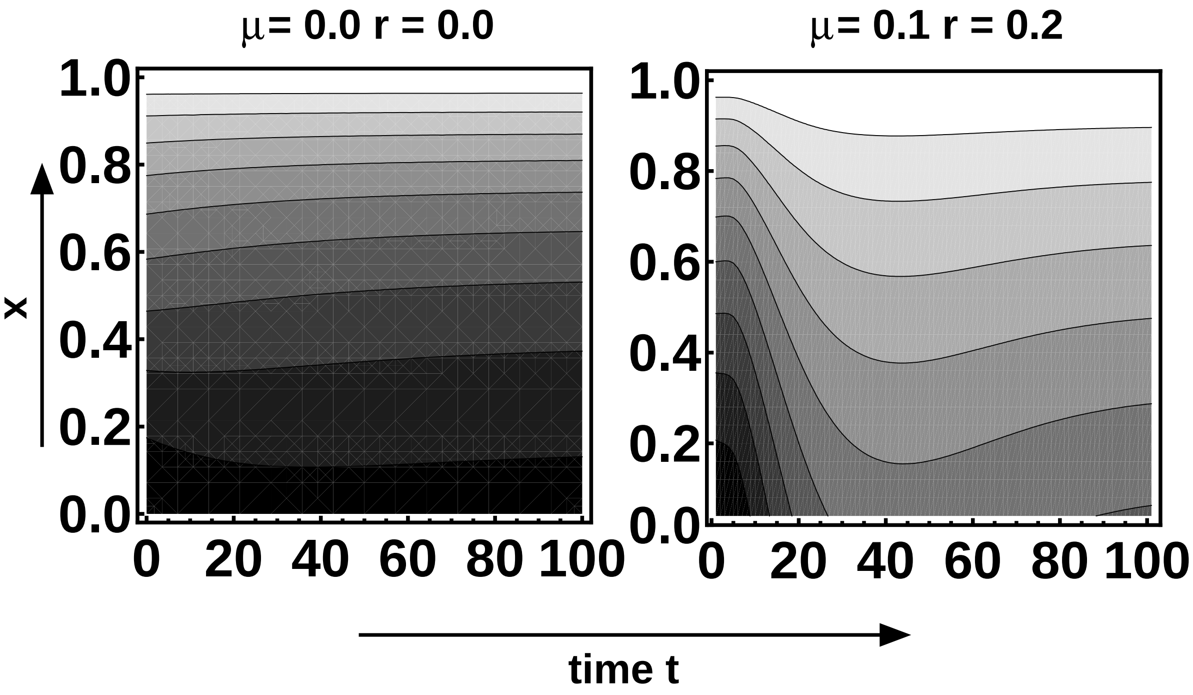

Figure 3: Time evolution of the probability generating function $g(x,t)$ for a network that originally has a scale free degree distribution of $p_k = \frac{k^{-\gamma}e^{-\frac{k}{\kappa}}}{Li_\gamma\left(e^{-\frac{1}{\kappa}}\right)}$ ($\gamma = 1.615$, $\kappa = 20$) with an average number of contacts per node of $\langle k \rangle$ = 3. Nodes enter and leave the network with the original distribution, i.e, $\bar{g}(x,t) = g(x,0)$ at a rate of η. Contour plots interpolate between 0 and 1 lightening up in steps of 0.1. The left panel shows the time evolution of $g(x,t)$ without an epidemic process; the right panel shows $g(x,t)$ under an epidemic with $\mu = 0.1$ and $r = 0.2$ (for details cf. [4]).

Figure 3 shows that the topological change observed in the total network can largely be attributed to the interaction with the epidemic process. To study whether this also applies to nodes of different age, a, we further analyze the contact behavior of nodes, taking into account their age. For this case study, we restrained our analysis to the simplest demographic scenario of identical birth and death rates, independent of time t and age a, i.e., $\eta = \eta_1 = \eta_2$ and in consequence a constant population size of $\bar{N}$. The population density $N(a,t)$ with respect to age is described by the following equation [5]

$$\frac{\partial}{\partial a}N(a,t) + \frac{\partial}{\partial t}N(a,t) = -\eta N(a,t) \tag{64}$$

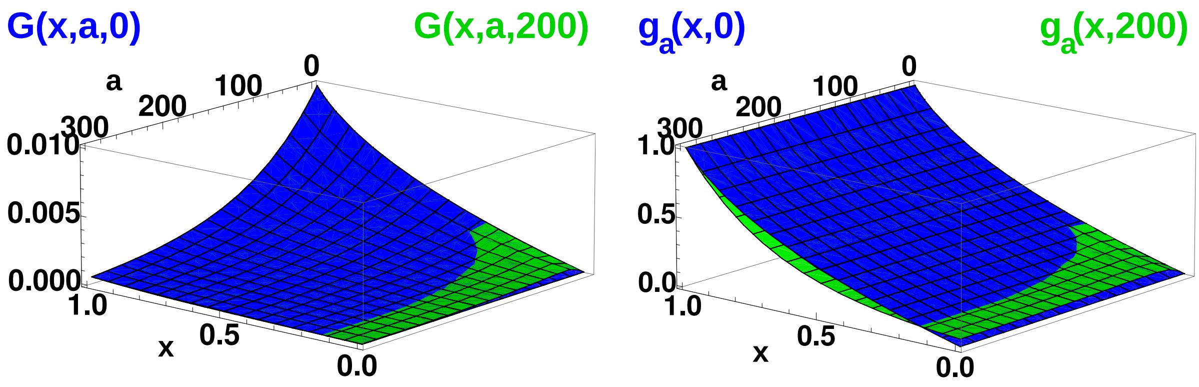

Figure 4: $G(x,a,t)$ and $g_a(x,t)$ at the initial stage $t=0$ (blue) and $t=200$ (green) of a network that originally has a scale free degree distribution $p_k = \frac{k^{-\gamma}e^{-\frac{k}{\kappa}}}{Li_\gamma\left(e^{-\frac{1}{\kappa}}\right)}$ ($\gamma = 1.615$, $\kappa = 20$) with an average number of contacts per node of $\langle k \rangle = 3$. Nodes enter and leave the network with the original distribution, i.e., $\bar{g}(x,t) = g(x,0)$ at a rate of $\eta = 0.01$. Differences mainly arise at $G(0,a,t) = \frac{N_0(a,t)}{\bar{N}}$ and $g_a(0,t) = \frac{N_0(a,t)}{N(a,t)}$, respectively. Although the original network does not have nodes without contacts, i.e., $g(0,0) = \bar{g}(0,t) = \bar{p}_0 = 0$, nodes without contacts are introduced by the demographic process. In the case of a Poisson degree distribution with same average degree, $G(x,a,0)$ (resp. $g_a(x,0)$) and $G(x,a,200)$ (resp. $g_a(x,200)$) are indistinguishable (data not shown).

in which the number of individuals between the ages of a_1 and a_2 is given by the integral of $N(a,t)$ over $[a_1,a_2]$. With initial and boundary conditions of $N(a,0) = \bar{N}\eta e^{-\eta a}$ and $N(0,t) = \eta\bar{N}$, this results in a population with a stable exponential age distribution, i.e., $N(a,t) = \bar{N}\eta e^{-\eta a}$ [5].

The population density can also be studied for individuals with a certain number of contacts, k, i.e., for $N_k(a,t)$, resulting in

$$\begin{aligned}\frac{\partial}{\partial a}N_k(a,t) + \frac{\partial}{\partial t}N_k(a,t) &= -\eta N_k(a,t) + \eta\frac{\partial}{\partial x}\bar{g}(1,t)(N_{k-1}(a,t) - N_k(a,t)) \\ &\quad -\eta(kN_k(a,t) - (k+1)N_{k+1}(a,t)).\end{aligned} \tag{65}$$

Assuming a constant total population, $N(t) = \int N(a,t)da = \bar{N}$, we further define

$$G(x,a,t) = \sum_k \frac{N_k(a,t)}{N}x^k = \frac{1}{\bar{N}}\sum_k N_k(a,t)x^k \tag{66}$$

which changes according to

$$\frac{\partial}{\partial a}G(x,a,t) + \frac{\partial}{\partial t}G(x,a,t) = -\eta G(x,a,t) + \eta(1-x)\frac{\partial}{\partial x}\bar{g}(1,t)G(x,a,t) + \eta(1-x)\frac{\partial}{\partial x}G(x,a,t). \tag{67}$$

This equation can be solved with the initial and boundary conditions $G(x,a,0) = \eta e^{-\eta a}\bar{g}(x,0)$, $G(1,a,t) = \eta e^{-\eta a}$ and $G(x,0,t) = \eta\bar{g}(x,t)$. Note that the probability generating function $g_a(x,t)$ for the conditional probability to have k contacts at age a is given by

$$g_a(x,t) = \sum_k \frac{N_k(a,t)}{N(a,t)}x^k = \frac{\bar{N}}{N(a,t)}\sum_k \frac{N_k(a,t)}{\bar{N}}x^k = \eta e^{\eta a}G(x,a,t). \tag{68}$$

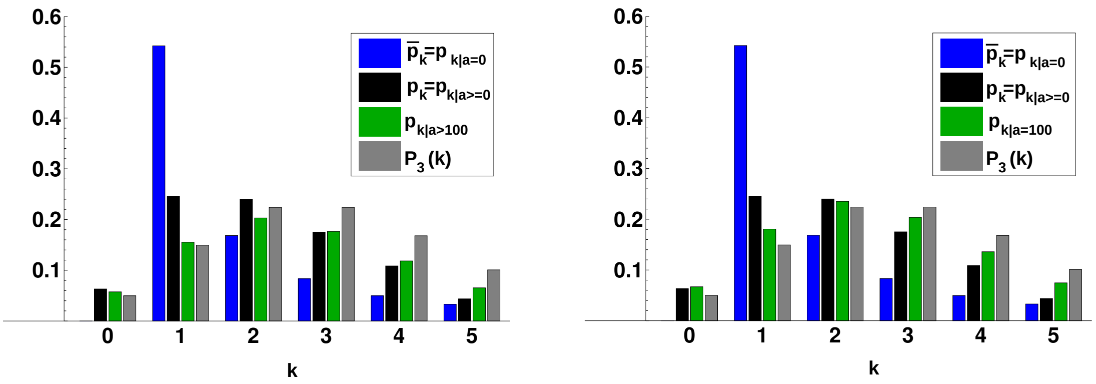

Figure 5: Time evolution in the degree distributions for several sub-populations at $t = 100$: The degree distribution of the original network and of newly entering individuals $\bar{p}_k = \frac{k^{-\gamma} e^{-\frac{k}{\kappa}}}{Li_\gamma\left(e^{-\frac{1}{\kappa}}\right)}$ ($\gamma = 1.615$, $\kappa = 20$, blue), the current degree distribution in the network p_k (black), the current degree distribution of individuals (at least) of age 100 (green) in comparison to a Poisson distribution with average $\langle k \rangle = 3$ (grey) are shown.

The probability generating function $g_{>a}(x,t)$ for the conditional probability to have k contacts, given a minimal age of a, can be written as:

$$g_{>a}(x,t) = e^{\eta a} \int_a^\infty G(x,a',t)da'. \tag{69}$$

Figures 4 and 5 demonstrate the age dependent topological consequences of the demographic process for a network that originally has a scale free degree distribution. Although the average degree is constant for all age groups, there are differences in the distributions of contacts. Newly introduced nodes show a heterogeneous contact pattern that becomes more centered on the average number of contacts with increasing age. However, the degree distribution of the total population does not converge over time towards a Poisson distribution. As a consequence of the presence of a few highly connected nodes, the distribution of contacts in the total population has a higher variance than expected for a Poisson distribution with the given average degree (data not shown).

Agent-based simulations

Agent based simulations were performed using NetLogo V4.0.4 [6], some code fragments were used from the model "Virus on Network" that is included in the software's model library [7]. Each simulation starts with the generation of a random network with a certain degree distribution followed by the simulation of the epidemic process on this network.

Two methods have been used to generate the initial random network:

- Generation of Poisson networks

  Poisson networks defined by a degree distribution $p_k = \frac{\langle k \rangle^k e^{-\langle k \rangle}}{k!}$ were generated by assigning $\langle k \rangle N/2$ links between randomly chosen nodes [8].

- Generation of random networks with a given degree sequence

  All random networks, except for Poisson networks, were generated from their degree sequence using the algorithm described in [9].

After infecting an initial fraction of random nodes, the simulation of the epidemic process on the random network is done according to the following pseudo-code until the epidemic dies out:

For each simulation step

1. each infected node progresses with a probability of μ (or μ_1 and μ_2, respectively) to the next infected stage (or death),
2. each infected node spreads disease to each of its susceptible contacts with a probability of r (or r_1 and r_2, respectively),
3. each node gives birth to a new node with a probability of η_1 and k contacts are randomly connected to already present nodes with a probability of $\bar{p}_k$,
4. each node dies with a probability of η_2 (including its links),
5. for each (undirected) link another random (undirected) link is chosen with a probability of $\rho/2$ after which the nodes at their ends are swapped (note that $\rho/2\times$ number of (undirected) links $\times$ number of changed (directed) contacts per swap/number of directed contacts $=\rho$).

Note that all parameters x (μ, μ_1, μ_2, r, r_1, r_2, η_1, η_2 and ρ) were scaled to be sufficiently small to satisfy $1 - e^{-x} \approx x \ll 1$ for each simulation step to comply with constant rates of progression/exponentially distributed waiting times in each compartment. In the exploratory studies, we found that there was a good trade-off between simulation precision and simulation time if the probabilities for transmission and recovery per simulation step are 0.02 and 0.01, respectively (other parameters scaled accordingly, cf. Fig. 1 of the main manuscript). The parameters in the simulations on HIV epidemics in Fig. 2 of the main main manuscript were scaled analogously, i.e., r_1, r_2, μ_1 and μ_2 were translated to probabilities of 0.0276, 0.001, 0.041 and 0.0012 per simulation step, respectively. This corresponds to simulation time steps of 0.01 years. While the computation time for the partial differential equations took seconds or minutes on a current typical desktop computer, the computation time for agent-based models took hours per run, i.e., days for a reasonable sample size.

References

[1] House T, Keeling M (2010) Insights from unifying modern approximations to infections on networks. *J. R. Soc. Interface*, e-pub. ahead of print.

[2] Volz, E (2008) SIR dynamics in random networks with heterogeneous connectivity. *J.Math.Biol.* 56:293–310.

[3] Volz, E, Meyers, LA (2007) Susceptible-infected-recovered epidemics in dynamic contact networks. *Proc.Biol.Sci.* 274:2925–2933.

[4] Kamp, C (2010) Demographic and behavioural change during epidemics. *Proc. Comp. Sci.* 1:2247–2253.

[5] Hoppensteadt, F (1975) Mathematical Theories of Populations: Demographics, Genetics and Epidemics. *SIAM CBMS-NSF Regional Conference Series in Applied Mathematics* pp. 1–15.

[6] Wilensky, U (1999) NetLogo. http://ccl.northwestern.edu/netlogo/, Center for Connected Learning and Computer-Based Modeling, Northwestern University. Evanston, IL.

[7] Stonedahl, F, Wilensky, U (2008) NetLogo Virus on a network model. http://ccl.northwestern.edu/netlogo/models/VirusonaNetwork. Center for Connected Learning and Computer-Based Modeling, Northwestern University, Evanston, IL.

[8] Durrett, R (2007) *Random graph dynamics* (Cambridge University Press).

[9] Molloy, M, Reed, B (1995) A critical point for random graphs with a given degree sequence. *Random Structures and Algorithms* 6:161–180.
